# Reopening California: Seeking robust, non-dominated COVID-19 exit strategies

**DOI:** 10.1371/journal.pone.0259166

**Published:** 2021-10-26

**Authors:** Pedro Nascimento de Lima, Robert Lempert, Raffaele Vardavas, Lawrence Baker, Jeanne Ringel, Carolyn M. Rutter, Jonathan Ozik, Nicholson Collier

**Affiliations:** 1 RAND Corporation, Santa Monica, CA, United States of America; 2 Pardee RAND Graduate School, Santa Monica, CA, United States of America; 3 Argonne National Laboratory, Lemont, IL, United States of America; University of Defence in Belgrade, SERBIA

## Abstract

The COVID-19 pandemic required significant public health interventions from local governments. Although nonpharmaceutical interventions often were implemented as decision rules, few studies evaluated the robustness of those reopening plans under a wide range of uncertainties. This paper uses the Robust Decision Making approach to stress-test 78 alternative reopening strategies, using California as an example. This study uniquely considers a wide range of uncertainties and demonstrates that seemingly sensible reopening plans can lead to both unnecessary COVID-19 deaths and days of interventions. We find that plans using fixed COVID-19 case thresholds might be less effective than strategies with time-varying reopening thresholds. While we use California as an example, our results are particularly relevant for jurisdictions where vaccination roll-out has been slower. The approach used in this paper could also prove useful for other public health policy problems in which policymakers need to make robust decisions in the face of deep uncertainty.

## Introduction

The COVID-19 pandemic has wreaked havoc across the US, causing over 37 million confirmed cases and 600,000 deaths, and state-wide shutdowns of all non-essential businesses. As state officials navigate the pandemic, they must manage both health and economic goals. Generally, reopening plans [1–4] have been proposed as phased strategies, in which the state allows economic activity to resume based on meeting COVID-19 incidence targets. However, pandemics often follow oscillatory waves of infection, and many states have already been forced to revise their plans, either shutting down after new outbreaks or adjusting which activities are allowed in each phase. To ensure long-term success in combating the pandemic, local policymakers need to consider the trade-offs underlying reopening decisions, while accounting for deep uncertainties.

Since one-time lockdowns have proven to be insufficient to control the pandemic, a coherent, long-term strategy is needed. Instead of adopting a stable, pre-defined strategy, local policymakers have changed regulations and instated NPIs adaptively, often adopting NPIs based on the decisions of other jurisdictions [5] without necessarily supporting every deliberation with thorough analyses. A key challenge in recommending a stable, transparent strategy—i.e., a clear prescription of which NPIs local policymakers should enact given a set of observed conditions—is to account for the effects of the biological, behavioral, and technological uncertainties surrounding the pandemic. One year into the pandemic, many factors and constraints are still unknown and outside of the control of policymakers, including the behavioral response to vaccination (i.e., change in population mixing behaviors after vaccination), vaccine uptake or vaccine efficacy to prevent transmission [6]. Variant strains with higher transmissibility [7] might also hamper the effects of social distancing measures and could impact vaccine efficacy, making it difficult to define and commit to a defined long-term strategy.

Model-based analyses of strategies for defending society against the COVID-19 pandemic have been invaluable. Prior studies evaluating how local governments should manage NPIs in the absence of COVID-19 vaccines have presented a grim outlook [8–11] justifying the need for stringent NPI strategies, and have revealed the impracticality of strategies solely based on naturally-acquired herd immunity [12]. Analyses specifically focusing on vaccine strategies accounting for age-dependent mortality and vaccine scarcity [13–16] have generally supported ACIP’s vaccine allocation recommendations [17] if one seeks to minimize deaths. More recently, analyses focusing on how to relax NPIs in the presence of vaccination generally find that premature reopening would result in resurgences and potentially compromise the benefits of vaccination [18–23].

This paper offers three contributions to the emerging literature of NPI strategies in the presence of COVID-19 vaccines. First, we evaluate 78 adaptive strategies to manage NPI levels, including strategies that resemble reopening plans with fixed thresholds such as California’s Blueprint for a Safe economy [3] and CDC’s Operational Strategy for Reopening Schools [24] as well as alternative strategies that change those thresholds over time. Adaptive reopening strategies based on case thresholds have been widely used to control the COVID-19 pandemic [1, 2, 4], often without being preceded or followed by decision analyses. Second, we consider economic consequences of NPIs, allowing the analysis of robustness trade-offs among strategies considering COVID-19 deaths, days of NPIs and income loss induced by NPIs. Third, we employ the Robust Decision Making (RDM) approach [25–27] to facilitate comparison of alternative adaptive NPI strategies and to address the deep uncertainties [28] surrounding the COVID-19 pandemic and vaccination rollout. Although RDM and other Decision Making Under Deep Uncertainty (DMDU) [29] methods have proven useful in other policy areas where uncertainties abound (i.e., climate change [30, 31], coastal resilience [32], terrorism insurance [33], water resources management [34], and transportation [35]), only few applications have used them for infectious disease modeling [36, 37], and we are not aware of other applications that informed COVID-19 policies and reopening plans.

RDM provides a framework designed to evaluate adaptive policies under deep uncertainty [26]. This framework builds on key concepts and tools from Decision Analysis, Assumption-Based Planning, Scenarios, and Exploratory modeling and is designed to provide decision making support under deeply uncertainty [27]. This paper employs RDM to stress-test COVID-19 reopening strategies that were implemented using adaptive components but still lack formal evaluation. For example, California’s reopening plan [3] and other reopening plans [1, 2, 4] are adaptive strategies that instate Nonpharmaceutical interventions in response to COVID-19 cases and other measures. However, these plans have changed over time, often without rigorous analyses preceding those changes. This paper includes specific recommendations for COVID-19 reopening policies and also demonstrates how one can use RDM to support policy decisions amidst an evolving public health crisis. RDM and other Decision Under Deep Uncertainty methods have been thoroughly discussed in the literature, and interested readers can refer to the seminal and recent RDM publications for those discussions [25, 27].

In this paper, we demonstrate that the design of those reopening plans shape the tradeoffs that these jurisdictions face. Our analysis demonstrates that holding reopening plans fixed can be a dominated strategy and make society unnecessarily worse off. That is, reopening strategies with fixed case thresholds can be pareto-dominated by strategies with time-varying or endogenous thresholds. Pareto-dominated strategies are those that are outperformed by another strategy in all criteria under consideration. Because pareto-dominated strategies makes society unnecessarily worse off, they should always be avoided. Our analysis and approach provides a framework for evaluating COVID-19 adaptive reopening strategies rigorously, and can support the improvement of reopening plans in the wake of vaccination roll-out. These results are relevant not only for California given its reopening plan [38], but also for countries or jurisdictions where vaccines are scarce. More broadly, these results are relevant to inform the response to future pandemics that may require similar responses.

The paper is organized as follows. First, the methods section provides a high-level overview of our approach, and details on the model can be found in our prior work [10, 39] and in the S1 Appendix document. Once the problem framing and our terminology is defined, the results section focuses on the main substantive finding that strategies with fixed thresholds might be dominated by strategies with time-varying thresholds. Finally, we discuss how improved strategies might be implemented, as well as potential challenges in pursuing pareto-efficient strategies.

## Methods

### Problem framing

State public health departments need to decide how to manage their NPI levels using a coherent set of rules that seek to minimize both the health and economic impacts of the pandemic. California’s Blueprint for a Safer Economy plan [3], for example, requires the number of daily new cases to be below 7 cases / 100 k and positivity rates to be below 8% to allow counties to move below their most stringent NPI level (widespread). As of Feb 1st, 2021, 99% of the state population was living under the widespread risk level, in which some non-essential businesses are closed or operate under restrictions. We frame the decision problem as to how to define the threshold criteria in those plans over time seeking to balance health and other welfare goals.

Reopening plans are not only defined by threshold criteria but also contain a larger set of decisions not analyzed in this paper. These decisions include which businesses are allowed to operate under each risk level, capacity constraints, and a set of adaptation measures. These decisions directly affect societal welfare by imposing differential costs on specific activities and have evolved over time within California’s reopening plan [3]. While examining these decisions could reveal that targeted interventions have the potential to produce pareto-improvements [40], doing so is beyond the scope of this paper. Therefore, this paper holds the definition of the risk levels constant and asks *how* one should navigate between the risk levels over time.

Although states can mandate low-cost mitigation measures such as voluntary social distancing and mask-wearing that have proven effective [41], imperfect compliance still allows the virus to circulate among the population in many US states. Therefore, the key decision-making challenge this paper investigates is how to manage blunt NPIs (such as restricting economic activity or interrupting in-person education), contingent on the net effect of other low-cost measures such as mask-wearing, behavioral changes, and physical adaptation measures. High compliance with low-cost measures and swift initial lockdowns, followed by tight strict movement interventions (e.g, New Zealand’s strategy), and appropriate levels of testing, contact tracing, and isolation to prevent re-seeding can potentially eliminate COVID-19 locally and obviate the need for this decision-making process and reopening plans. In practice, however, US states and many countries have failed to control the spread of the virus through those instruments, thus forcing local decision-makers to still face trade-offs and impose restrictions.

When crafting reopening plans, decision-makers should seek strategies that are *both robust and non-dominated*. A non-dominated strategy (also called pareto-efficient) is one for which it is not possible to improve one relevant societal outcome (e.g. health) without making worse some other societal outcome (e.g. economic income). A robust strategy is one that performs well compared to the alternatives across a wide range of futures. A focus on non-dominated strategies provides decision-makers with a set of best-possible tradeoffs among societal objectives, without pre-judging how much decision-makers ought to weigh one objective over another. A focus on robust, non-dominated strategies aims to provide decision-makers with a set of tradeoffs that are non-dominated irrespective of how various uncertainties in fact unfold.

While seeking robust, non-dominated policies, decision-makers have to consider a number of factors, including the goals that one seeks to achieve, the policy levers that are within reach, uncertainties that can influence the decision between those policy levers, and how those elements are connected. The next sections describe the main elements of this decision problem that are included in our analysis, using an XLRM framework [26]. The letters X, L, R, and M refer to four categories of factors important to the analysis: outcome measures (M) that reflect decision makers’ goals; policy levers (L) that decision-makers use to pursue their goals; uncertainties (X) that may affect the connection between levers and outcomes; and relationships (R), instantiated in the simulation model, that link uncertainties and levers to outcomes. The subsequent sections provide details on each of those four basic elements.

### Relationships

This paper builds from the models underlying RANDs COVID-19 State Policy Tool [10, 39, 42]. The original model and tool use a state-level deterministic epidemiological model [39] and a general equilibrium economic model [42] to inform state policymakers on the trade-offs of alternative NPIs. The set of NPIs used by each US state is characterized by a discrete set of intervention levels ranging from 1 (no intervention) to 6 (close schools, bars, restaurants, and nonessential businesses; and issue a shelter-in-place order for everyone but essential workers). The economic model [42] provides an estimate of weekly income loss for each US state and each intervention level, which we integrate over time based on the NPI levels used in the epidemiological model. The epidemiological model contains five strata: those below 18 years (Young), those with more than 65 years with or without chronic conditions, frontline essential workers, working-age individuals with chronic conditions, and working-age individuals without chronic conditions. Each intervention level is associated with population mixing matrices that describe how strata interact with each other in six different settings: household, work, school, commercial, recreation, and other [10, 39]. Interventions are modeled as changing the level of mixing which occurs in each of these settings. For instance, closing schools reduces school and work mixing but increases home mixing. Given the specified model structure, the NPI time series, and the mixing matrices, the model can be calibrated and run for any US state using cumulative monthly deaths time series [43]. In this analysis, the model was calibrated in the period of March 1st 2020 through Dec 25th 2020 for the state of California. The policies are evaluated from Dec 25th through Jan 31st, 2021. Further details about our model can be found in the S1 Appendix.

### Policy levers and strategies

Following the definitions commonly used in state-level plans, we define NPIs as alternative sets of intervention levels. Within each NPI level, a set of restrictions are imposed. For instance, when *NPI*_*t*_ = 1, the lowest level, businesses and schools are opened, although society may still have inexpensive policies in place, such as mask-wearing. When *NPI*_*t*_ = 6, the highest level, society imposes the most stringent restrictions on people leaving their homes and interacting with others.

An NPI Strategy consists of a rule that determines how NPI levels change over time. The time-dependent NPI level can be specified as a function of COVID-19 prevalence *p*_*t*_ using the controller function:

$$\begin{array}{c} NPI_{t}^{*}=\{\begin{array}{cc} \text{min}(x_{t}^{s}p_{t}\times 10^{3},5)+1 & \text{if}\;V_{t}<V^{*}\\ 1 & \text{if}\;V_{t}\geq V^{*} \end{array} \end{array}$$ (1)

where *p*_*t*_ represents estimated COVID-19 prevalence scaled by 10^3^, and $x_{t}^{s}$ represents the sensitivity of policymakers to this prevalence which we label “level of caution”, *V*_*t*_ is the current vaccination coverage rate, and V* is the threshold vaccination coverage rate at which policymakers terminate the use of NPI’s. The controller function is bound between one and six, corresponding to the six intervention levels in our model. This results in a policy wherein a $x_{t}^{s}0.1\%$ increase in COVID-19 prevalence results in policymakers increasing restriction levels by one level until the highest restriction level is adopted. This controller function provides a simple representation of the types of phased reopening plans used by US states, such as California’s Blueprint for a Safer Economy plan [3]. Eq 1 also reflects the fact that policy-makers might exhibit different levels of sensitivity to changes in COVID-19 prevalence, and that this sensitivity $x_{t}^{s}$ can change over time.

In this study, we ask how $x_{t}^{s}$ should be managed over time. We evaluate three types of NPI Strategies, denoted by *s* = *C*, *T*, and *V*, which differ in how the level of caution $x_{t}^{s}$ is managed. The strategies can use a constant level of caution (*s* = *C*), a two-step function of time (*s* = *T*), or a smooth function of the proportion of the population that is vaccinated (*s* = *V*). We define each type of strategy as follows:

$$\begin{array}{c} x_{t}^{s}=\{\begin{array}{cc} x_{b}, & \text{if}\;s=\text{C}\\ x_{b}, & \text{if}\;s=T\;\text{and}\;t<T_{\alpha }\\ \alpha x_{b}, & \text{if}\;s=T\;\text{and}\;t\geq T_{\alpha }\\ x_{b}(1-\frac{1}{1+e^{-k_{c}\left(V_{t}-V_{mid}\right)}}), & \text{if}\;s=V \end{array} \end{array}$$ (2)

The constant level of caution strategy (*s* = *C*) holds $x_{t}^{s}$ constant at a baseline level of caution *x*_*b*_. The two-step strategy time-based strategy (*s* = *T*) begins with the value *x*_*b*_ and changes to *αx*_*b*_ at a predefined date *T*_*α*_ (i.e., *T*_*α*_ could be the beginning of spring, or the fall). We assume, that and 0 > *α* > 1. The vaccination-based strategy (*s* = *V*) calculates a time-varying level of caution as a smooth function of the cumulative number of persons fully vaccinated *V*_*t*_ using an inverse logistic function that starts at 1 when *V*_*t*_ = 0, and ends at zero when *V*_*t*_ = 1, with mid-point *V*_*mid*_, and curvature defined by *kc*. A particular NPI strategy is defined by the choice of values for the parameters *s*, *x*_*b*_, *α*, *V*_*mid*_, and *k*_*c*_.

**Fig 1:**
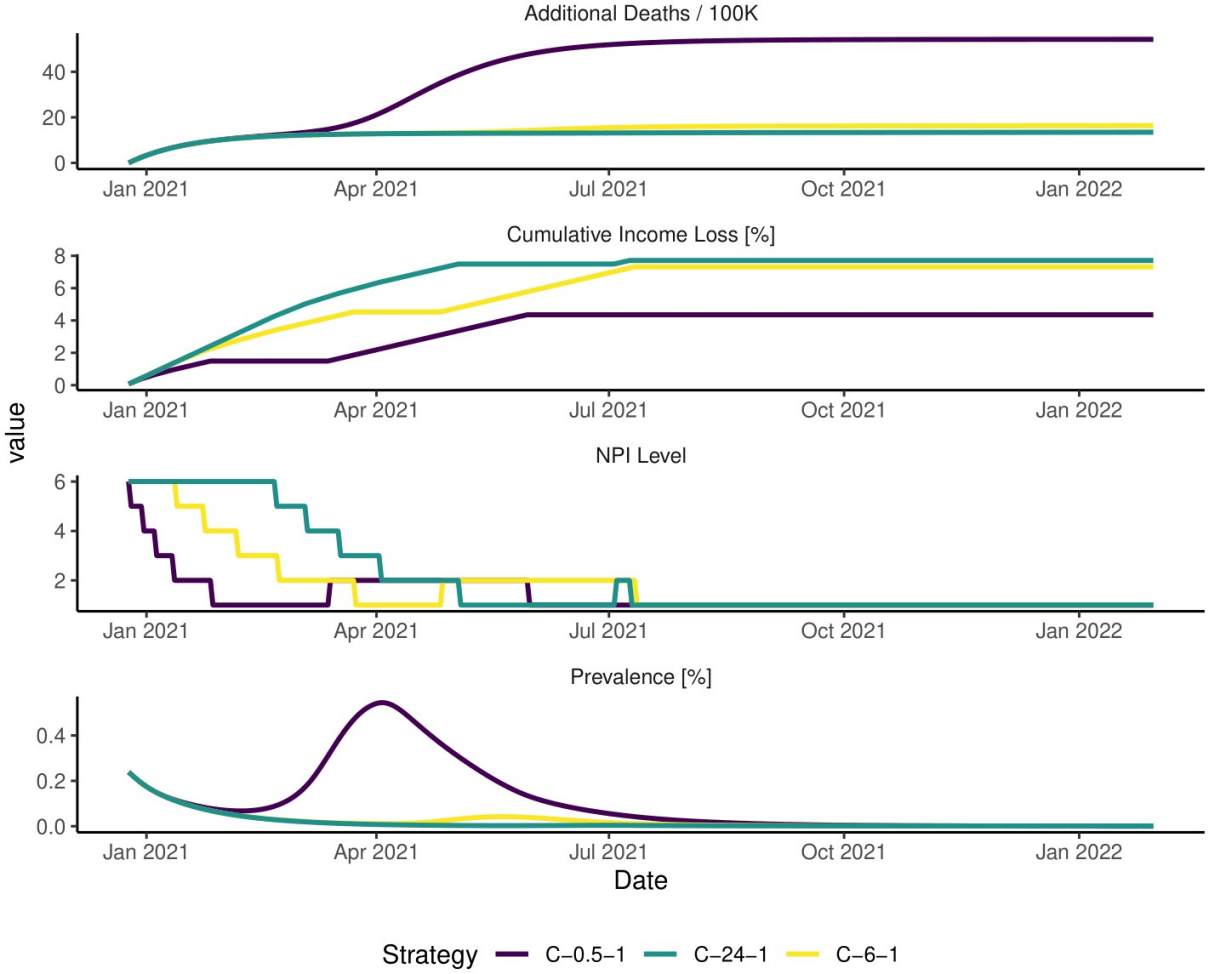
Model dynamics with adaptive NPI strategies in California. This figure presents three illustrative runs testing three strategies in an example future state of the world. Strategies are coded as follows throughout this paper. The first letter in the strategy code represents the strategy type (C for constant caution, T for time-based, and V for vaccine-based strategies). The subsequent number represents the baseline level of caution *x*_*b*_, and the third number is a sequential code to make the strategy code unique. The full list of strategies tested in this paper is provided in S1 Appendix. In this figure, the most stringent strategy, C-24-1 uses a level of caution *x*_*t*_ = 24 throughout the simulation run. Strategy C-6-1 uses a level of caution *x*_*t*_ = 6 and produces similar results, while strategy C-0.5-1 results in a substantial increase in deaths. None of these model runs represent a forecast.

Fig 1 illustrates the model dynamics with a constant level of caution *x*_*t*_. This formulation leads to frequent interventions, similar to patterns seen in California. Note that with a low level of caution (*x*_*b*_ = 0.5) prevalence is allowed to increase substantially before a state takes action. With a high level of caution (*x*_*b*_ = 24) prevalence is held much lower. As a reference, California’s criteria of 7 cases / 100 thousand people to relax the most stringent intervention level translates to a level of caution *x*_*t*_ ≈ 5 to 5 in our model. One can translate a case rate threshold *c* to our model’s level of caution *x* by approximating the known prevalence *p* ≈ *c* * *τ* from Little’s Law [44], where *τ* is the average duration of the disease and *c* is the case rate criteria, and *p* represents an estimate of the prevalence. From that, and considering our model has five intervention levels, we can translate any case rate criteria to a level of caution by letting *x* = 5/(c*τ*10^3^), which leads to a level of caution of five when *c* = 7/100*k* and *τ* = 14. We defined the range of the baseline level of caution parameter *x*_*b*_ by exploring the tradeoff surfaces they produce and ensuring that the range includes the edges of the tradeoff space presented in Fig 1. We do not include a level of caution of 0 in our analysis because we regard this level of caution as unrealistic.

Eqs 1 and 2 represent a range of alternative strategies to manage NPIs during vaccination roll-out. If California were to change its thresholds and reduce restrictions in the fall of 2021, that could be represented by a time-based strategy *s* = *T* with a baseline level of caution *x*_*b*_ = 5, transition time *T*_*α*_ = *Fall*, 2021 and a reduction factor of *α* = 0.5. If *α* = 0, California would allow businesses and in-person education to operate at *T*_*α*_. Similarly, California might decide to slowly scale down restrictions based on the number of vaccinations, such that restrictions would be halved when 50% of the population is vaccinated. This policy would be represented by a strategy *s* = *V*, with *x*_*b*_ = 5 and *V*_*mid*_ = 0.5. We consider a set of strategies by combining strategy types and their parameters in a full-factorial experimental design. The full list of strategies and the parameters used in this paper is provided in the S1 Appendix, and a sub-set of the 78 strategies is presented in Table 1.

**Table 1:** Regret percentiles by metric and strategy for a set of non-dominated and constant-caution strategies.

Strategy	Strategy Parameters	Deaths per 100 k	NPI Days
C-24-1	constant	0	252
V-24-2	V* = 60%; *k*_*c*_ = 15	1	248
T-24-6	*α* = 50%; *T*_*α*_ = 2021 − 09 − 26	1	249
V-24-1	V* = 60%; *k*_*c*_ = 10	1	248
T-24-5	*α* = 50%; *T*_*α*_ = 2021 − 07 − 04	1	248
V-24-5	V* = 50%; *k*_*c*_ = 10	2	244
V-24-6*	V* = 50%; *k*_*c*_ = 15	3	240
T-24-4*	*α* = 50%; *T*_*α*_ = 2021 − 03 − 10	4	229
C-12-1	constant	4	247
T-24-3*	*α* = 10%; *T*_*α*_ = 2021 − 09 − 26	5	225
V-12-2	V* = 60%; *k*_*c*_ = 15	5	242
T-12-6*	*α* = 50%; *T*_*α*_ = 2021 − 09 − 26	5	238
V-12-1	V* = 60%; *k*_*c*_ = 10	5	243
V-24-3*	V* = 40%; *k*_*c*_ = 10	6	238
T-12-5*	*α* = 50%; *T*_*α*_ = 2021 − 07 − 04	6	240
V-12-5*	V* = 50%; *k*_*c*_ = 10	8	238
V-12-6*	V* = 50%; *k*_*c*_ = 15	8	232
T-24-2*	*α* = 10%; *T*_*α*_ = 2021 − 07 − 04	9	213
V-24-4*	V* = 40%; *k*_*c*_ = 15	10	222
T-12-4*	*α* = 50%; *T*_*α*_ = 2021 − 03 − 10	11	222
T-12-3*	*α* = 10%; *T*_*α*_ = 2021 − 09 − 26	12	217
**C-6-1**	**constant—(baseline strategy)**	**12**	**238**
T-6-6	*α* = 50%; *T*_*α*_ = 2021 − 09 − 26	14	223
V-6-2	V* = 60%; *k*_*c*_ = 15	14	231
V-6-1	V* = 60%; *k*_*c*_ = 10	15	230
T-6-5	*α* = 50%; *T*_*α*_ = 2021 − 07 − 04	16	224
V-6-5	V* = 50%; *k*_*c*_ = 10	19	218
V-6-6	V* = 50%; *k*_*c*_ = 15	19	213
T-6-3	*α* = 10%; *T*_*α*_ = 2021 − 09 − 26	22	204
T-6-4	*α* = 50%; *T*_*α*_ = 2021 − 03 − 10	24	211
V-6-3	V* = 40%; *k*_*c*_ = 10	27	195
C-3-1	constant	28	209
T-6-2	*α* = 10%; *T*_*α*_ = 2021 − 07 − 04	33	168
V-6-4	V* = 40%; *k*_*c*_ = 15	33	177
C-1.5-1	constant	50	161
T-6-1	*α* = 10%; *T*_*α*_ = 2021 − 03 − 10	71	112
C-0.5-1	constant	93	73

The table presents a subset of the strategies evaluated in this paper, and the full list of strategies is available in the S1 Appendix. This table presents only strategies that use a constant level of caution, or dominate the baseline strategy C-6-1, allowing for a margin of 1%. Strategies are coded as follows. The first letter in the strategy code stands for the strategy type (C for constant caution, T for time-based, and V for vaccine-based). The subsequent number represents the baseline level of caution *x*_*b*_, and the third number is a sequential number making the strategy code unique. The parameters column describes policy levers that characterize each strategy, as described in the Methods section. The final three columns present the 75^*th*^ regret percentile of three metrics of interest.

### Uncertainties

We categorize uncertainties into two distinct classes: well-characterized uncertainties and deep uncertainties. Well-characterized uncertainties are those for which historical data or clinical evidence can provide information, either directly or through calibration. Deep uncertainties [28] are those for which calibration or existing clinical evidence provides little information at the time of this writing, and have a high potential to affect the choice of the strategies.

Well-characterized uncertainties include the length of disease states, which are set to fixed values based on published findings, and parameters that are selected using model calibration, including the magnitude of the seasonal effect on mixing. Calibrated parameters are chosen using the Incremental Mixture Approximate Bayesian Computation (IMABC) approach [45]. The IMABC algorithm results in 100 simulated draws from the posterior distribution of the parameter set. This parameter set, hereafter termed “calibrated parameters”, contains values for 42 model parameters, including information about how effective NPIs have been in the past, mortality rates, and disease progression rates. Calibration priors, targets and the calibration process are discussed in the S1 Appendix.

Deep uncertainties include vaccine efficacy to prevent transmission, the behavioral mixing response to vaccination, willingness to vaccinate, changes in transmissibility, immunity duration, and the actual vaccination rate. Uncertainties surrounding vaccine efficacy are particularly concerning given their impact on the pandemic dynamics. While vaccine efficacy to prevent disease has been established, vaccine efficacy to prevent infection is unknown at the time of this writing [6, 46, 47]. Similarly, the effect of new variant strains on transmissibility and other disease parameters is of particular concern. For example, variants B.1.1.7, B.1.351, and P.1 have demonstrated an impact on transmissibility, inactivity, and antibody escape capabilities [7].

To examine the impact of deep uncertainties on future outcomes, we first draw a quasi-random sample of 200 unknown parameter vectors using Latin Hypercube sampling to ensure representation of the parameter space. Details about these parameters and their bounds are provided in S1 Appendix under the Experimental Design section. We then combine each row in our “calibrated parameters” dataset with each row in our Latin Hypercube to create a new dataset with 20,000 rows in which each row represents a single future state of the world (SOW). The final experimental design is obtained by testing each strategy in each of the 20,000 future states of the world. We define our set of strategies using a full-factorial experimental design yielding 78 strategies, described in S1 Appendix. Hence, our experimental design contains 1.56 million cases (78 strategies * 20,000 future states of the world). Following the RDM approach [25], we evaluate how each strategy would perform in a wide range of futures, and judge those strategies by their ability to cope with many potential futures. Readers unfamiliar with the RDM approach can think of our analysis as stress-test of each candidate policy across a wide range of plausible futures. Further details about the parameters included in the experimental design and justification for those decisions are are found in the S1 Appendix.

### Outcome measures

When judging alternative reopening strategies, policymakers often have to weigh multiple criteria to make decisions. Our epidemiological model computes the cumulative number of COVID-19 cases per 100,000 people including undetected cases, years of life lost due to COVID-19 deaths per 100,000 people, and COVID-19 deaths per 100,000 people. Our model does not simulate any health outcome that is not directly attributed to a COVID-19 infection, and does not include excess deaths other than COVID-19 deaths [39]. Reopening decisions, however, have far-reaching social welfare consequences which are not explicitly computed in COVID-19 epidemiological models. While short-term economic impacts might have been limited in some circumstances [48], the full social welfare cost of NPIs includes the effects of interrupted in-person education [49], mental health impacts of isolation, other illness exacerbated by reduced use of non-COVID health services, impacts of financial effects on mental and physical health, deaths of despair [50], and long-term loss of income. While we are not aware of a published estimation of the total welfare loss induced by NPIs, one needs to find criteria that can be computed from the model and are proportional to the marginal welfare loss induced by alternative NPI levels.

One proxy for the welfare cost of NPIs already used in prior analyses is the number of days under NPIs [8]. This criterion reflects the number of days that society needs to close non-essential workplaces, which included schools. Without loss of generality, this measure (number of days with *NPI*_*t*_ > 1) also reflects the number of days with school closures in this analysis. We adopt this proxy of social welfare cost because it has already been used by other analyses thus allowing us to compare results, it is easily interpreted, and require a minimal set of assumptions. The only assumption one needs to accept for using this criterion is that society is worse off when activities that would be otherwise allowed are interrupted, and that more time under interventions result in a higher social welfare cost. Thus, This is the outcome used in this paper to assess pareto-efficiency.

While it is easily interpreted, this outcome does not consider that more stringent NPI levels could imply more severe social welfare costs, so other proxies for welfare loss might be desirable. One approach is to use weights for each NPI level, such that those weights are proportional to the marginal daily welfare loss induced by each NPI level. With the purpose of demonstrating how this could be done, we use an estimate of Income Loss as those weights to present a proxy for economic consequences of NPI restrictions [42]. Although these proxies are imperfect measures of social welfare loss induced by NPIs, the conclusions in this paper not rely on their precision, but on the assumption that NPI costs are increasing in the level of restriction. This structural assumption allows us to illuminate trade-offs and reveal pareto-dominated strategies. Therefore, our findings do not rely on the precision of any welfare loss estimate, neither does it rely on the structure or assumptions of the economic model but only on the structure of the epidemiological model. Alternatively, one might estimate the costs of NPIs using a willingness to pay or a similar approach. As long as the resulting weights are monotonically increasing (i.e., people are not deriving utility from NPI restrictions), our substantive findings would hold because we refrain from aggregating measures. While estimating more precise welfare costs of NPIs and using those costs as a criterion could be valuable to compare benefits and costs at the same scale, we doubt that this approach would lead to precise estimates because these weights are likely not stable over time due to economic diversion [48] and adaptation. Still, as long as these weights are monotonically increasing in the NPI level *at any point in time* our substantive results would hold. Because the weights are highly uncertain, potentially not constant, and not necessary for the purposes of this analysis, we refrain from trying to aggregate all outcomes under a single social welfare metric in our analyses as a traditional Cost-Benefit analysis would. Instead, we assess pareto-efficiency and seek strategies that dominate other strategies across a set of outcomes, over a wide range of futures.

Because our interest lies in the robustness of strategies rather than in their optimality for any particular future, we use Regret [25] as a robustness metric and the 75^*th*^ Regret percentile as a decision criterion. Regret is computed for each metric of interest as follows. We construct a dataset of model runs in which each row contains the values of the outcomes defined above at the end of the simulation. Each row in this dataset represents the performance of each strategy on each future state of the world (SOW) characterized by uncertainties as described previously. Robustness in this study is operationalized with a separate regret metric for each outcome of interest. Regret *R*_*s*,*f*_ is defined for each strategy *s* in each SOW *f* as the difference between the observed outcome and the best possible outcome in that future:

$$\begin{array}{c} R_{s,f}=D_{s,f}-\underset{x}{\text{min}}\left[D_{x,f}\right] \end{array}$$ (3)

The goal of the decision-making process is to adopt a strategy that minimizes regret across a wide range of potential futures, across all of the outcomes of interest. When trying to minimize regret across a wide range of outcomes and futures, decision-makers often find that prioritizing a single set of outcomes imposes regret on other outcomes. Similarly, minimizing regret in a single future might create vulnerabilities to other future states of the world. There are multiple approaches to summarize the many-objective robustness trade-offs implied by alternative strategies when evaluated across a wide range of plausible futures. For example, if one chooses the 100^*th*^ percentile, this approach corresponds to a minimax regret criterion. If one chooses the mean and assumes that future states of the world are equally probable, that would correspond to a Laplace criterion. Here we choose the 75^*th*^ percentile. While changing this percentile can change the strategy rankings, doing so did not change the main substantive conclusions of this paper that constant-caution strategies were dominated. While a thorough discussion on the use of regret metrics is beyond the scope of this paper, the interested reader can refer to methodological pieces discussing the use of robustness metrics [25, 26, 51].

## Results

This section describes two main results from our analysis, namely: i) the trade-offs among the baseline and alternative strategies, and ii) the existence and characteristics of dominated policies. While traditional decision analyses often rule out dominated policies as a first step, we find it useful to focus our discussion on those strategies with the goal of illuminating potential alternatives.

**Fig 2:**
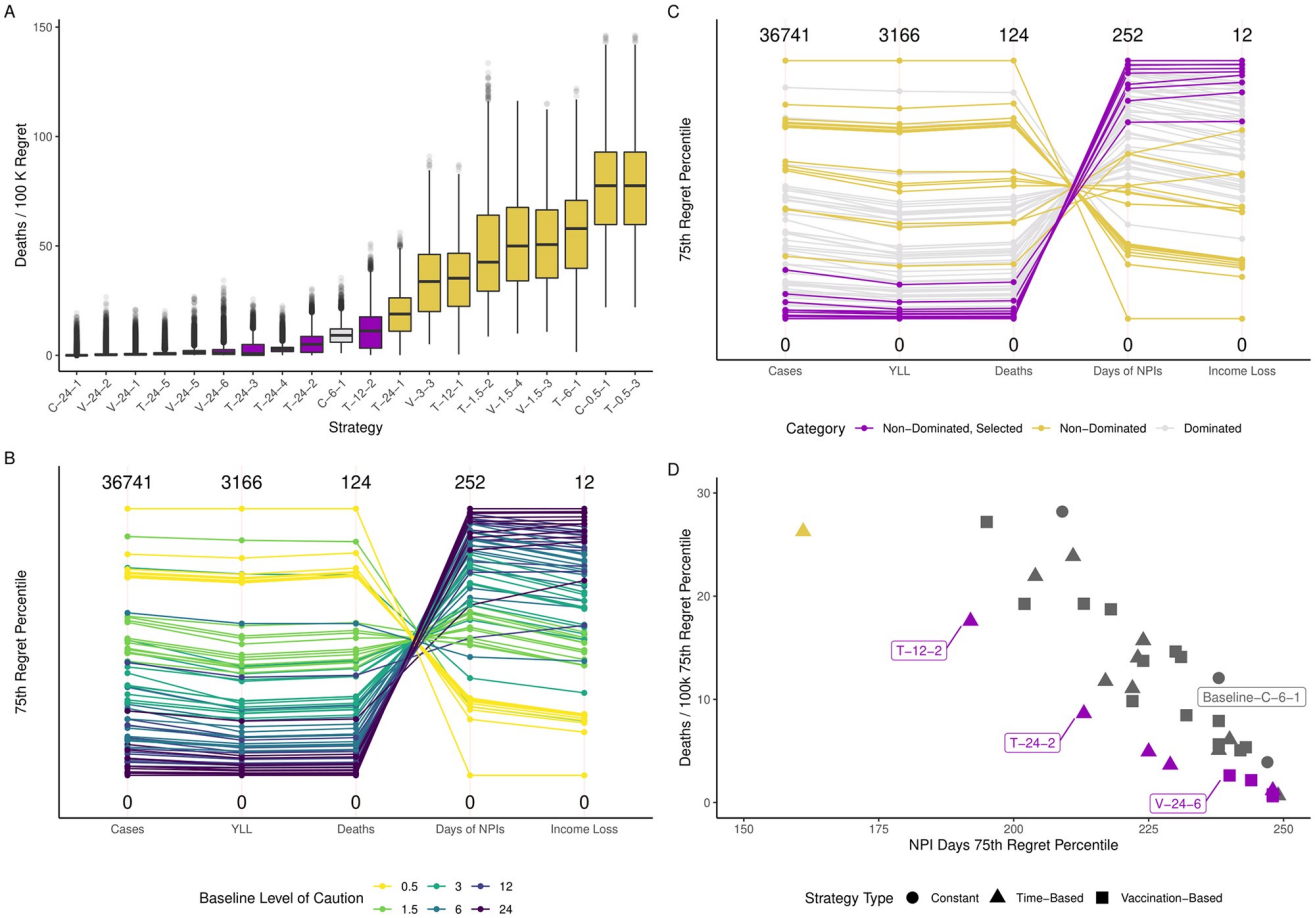
Robustness trade-offs emerging from 78 alternative reopening strategies. Panel A shows the distribution of a Regret metric for four outcomes of interest. Each line in Panel B and C represents a single strategy. Vertical axes represent the 75^*th*^ Regret percentile for each metric. All Health outcomes are normalized per 100 thousand people. Strategies are coded as follows. The first letter indicates whether the NPI strategy uses a Time-based level of caution (T), a Vaccination-Based level of caution (V), or a Constant level of caution (C). The subsequent number describes the baseline level of caution *x*_*b*_ and a third number is a sequential number that creates a unique code for each strategy. Table 1 contains a description of all strategies.

Fig 2(A) presents the distribution of Deaths / 100,000 people for a sub-set of the 78 strategies considered in this paper. These results emphasize that only strategies that use a high initial baseline level of caution (*x*_*b*_ ≥ 6) are able to control deaths and thus result in low regret in terms of deaths. The strategies using lower baseline levels of caution unsurprisingly result in higher death regret. These results demonstrate that the baseline level of caution non-linearly affects deaths. For example, Fig 2(B) demonstrates that there is a significant reduction in the number of deaths when one compares the level of caution 0.5 to 1.5, but using a level of caution of 24 instead of 12 marginally reduces deaths.

The baseline strategy with a fixed level of caution (C-6-1) seems to achieve a compromise when compared to more stringent strategies. More stringent strategies (i.e., C-24-1, C-12-1) are able to achieve a lower number of deaths regret, but doubling the level of caution does not halve the number of deaths, nor does it double the social welfare costs, as measured by Loss of Income or Days under NPIs. This result indicates that a baseline strategy with a level of caution *x*_*b*_ ≥ 6 is necessary to robustly control the number of deaths. If the goal of policymakers was only to minimize the number of deaths from the pandemic, these results would imply that the best approach would be to sustain the highest level of caution indefinitely.

Preventing deaths, however, comes with a cost. Fig 2(B) contains other outcomes produced by the set of strategies evaluated. In Fig 2B, each line represents one of the 78 tested strategies, and the parallel axes summarize the performance of each strategy using the 75^*th*^ percentile of the regret distribution shown in panel A for each one of the metrics. Again, this figure reveals that only strategies with higher baseline levels of caution (i.e., *x*_*b*_ > 3) are able to reach the bottom of the Deaths regret scale. Unsurprisingly, strategies that achieve the lowest numbers of deaths are also the ones that generate the highest social welfare costs, as measured by the two proxies available. Although the order of magnitude of the outcomes can vary among other models, this finding is in line with prior studies using multiple models [8].

Although the baseline strategy (C-6-1) seems to produce acceptable health outcomes given the other alternatives, that does not imply that this strategy is pareto-efficient. To assess pareto-efficiency, we categorize strategies into three classes. Non-dominated strategies are those that are not outperformed by any other strategy with respect to health and social welfare outcomes using the 75^*th*^ regret percentile as a criterion. Because all health outcomes are correlated, we use Deaths and Days under NPIs as criteria to perform pareto-sorting and determine which policies are dominated. We select strategies with relatively low numbers of deaths seeking to represent a decision-maker who is willing to prioritize health outcomes. Finally, dominated strategies are those that exhibit worse health or social welfare outcomes. These strategies are color-coded in panels C and D of Fig 2.

Fig 2(D) reveals a troubling pattern for jurisdictions pursuing strategies based on constant COVID-19 case thresholds. The figure illustrates that strategies with constant levels of caution (circles) are dominated by *many* other strategies. Vaccination-based strategies (squares) and time-based strategies (triangles) are closer to the origin in that plot. Table 1 includes the strategies with constant level of caution and flags strategies that dominate the baseline strategy (C-6-1) with an asterisk. We find that strategies using a constant level of caution (the circles in Fig 2D) are systematically dominated, except for the most stringent strategy or the most relaxed strategies. That is to say, unless society is willing to indefinitely sustain the highest level of stringency we considered, sustaining a fixed level of caution is always dominated by strategies that change the level of caution over time.

Of course, this result does not mean that California or other jurisdictions can start with a level of caution of six and achieve better results by lowering the same level of caution. As Table 1 indicate, any reduction of the level of caution necessarily results in higher deaths. For example, starting with a level of caution of six and reopening too soon (e.g., strategy T-6-4 starts with a level of caution of 6 and reduces the level of caution by 50% on March 10th, 2021) results in doubling the number of deaths 75^*th*^ regret percentile, while achieving only a decrease of 27 days of number of days under NPIs.

The strategies that dominate the baseline strategy (C-6-1) while resulting in a lower or equivalent number of deaths share the same characteristics. They start with a higher level of caution, then relax their level of caution as vaccination and time advances. For example, strategy T-12-5 starts with a higher level of caution of 12 and relaxes its level of caution by 50% on the fourth of July. By doing so, this strategy outperforms the baseline strategy C-6-1 by halving the number of deaths using two additional days under NPIs. Starting with high stringency levels then relaxing when immunity is widespread is arguably what other countries with higher capacity to control COVID-19 (i.e., New Zealand) will do and our results support that strategy. Although California’s Blueprint for a Safer economy plan originally had fixed thresholds, California has imposed a regional stay-at-home order from December 2020 through January 2021, effectively increasing the level of caution in that period. Recently, California updated its plan and made it dependent on vaccination rollout [52]. Our results generally support changes on reopening plans that effectively make reopening policies more stringent while immunity is not widespread. Nonetheless, our results also demonstrate that stress-testing a wide range of strategies against other alternatives is important to verify whether selected policies are not clearly dominated by other alternatives.

While simpler SEIR models with homogenous populations might produce similar results, that does not need to be the case. We attribute our results and the rationale behind the vaccine-based strategies to the interactions between the vaccination strategy being used and the heterogeneities included in our model. As more individuals from the most vulnerable groups are immunized first, the average infection-fatality ratio (IFR) amongst the currently infected should decrease, thus suggesting that strategies with a fixed level of caution would be dominated. In the limit, as we approach an endemic state [53], the marginal benefit of NPIs decrease and the strategies accounting for these dynamics dominate strategies with fixed thresholds. The time scale of that transition depends on several factors, including heterogeneous fatality rates, vaccination strategy, and the rate at which immunity increases and wanes in the population. Our analysis evaluated these policies not only on a single-best estimate of model parameters, or on a single sample from the posterior distribution of calibrated parameters, but against 20,000 futures defined by well-characterised and deep uncertainties (see S1 Appendix). Other analyses using similar models or agent-based models that incorporate those heterogeneities should reach similar conclusions.

## Discussion

Our main substantive conclusion is that adaptive reopening strategies with fixed thresholds can be dominated by alternatives that are more stringent but change their stringency over time. While this finding points to potentially better policies, it also demonstrates that seemingly sensible reopening policies might not be pareto-efficient. This finding has important implications for existing reopening plans, not only in California but also for other US states and countries pursuing similar reopening strategies. These findings suggest that localities with stringent policies might have to craft time or vaccine-based reopening policies as vaccination is made widely available. Similarly, jurisdictions pursuing strategies with a low baseline level of caution might be trading death regret for small benefits, and are vulnerable to the emergence of new, more transmissible strains. Failing to cautiously adjust reopening policies will result in excess deaths from premature reopening decisions and/or unnecessary economic burden on those most vulnerable. While balancing multiple outcomes has not generally been done formally by policymakers (i.e., we still fail to find reopening plans that are backed by many-objective analyses stress-testing the decision rules embedded in those reopening plans), these decisions are arguably the most important policy decisions of 2021 and have far-reaching consequences.

After demonstrating that current strategies can be pareto-dominated, the next question is whether and how pareto-efficient strategies can be implemented. This paper does not offer a fixed timetable for when thresholds should be changed because doing so would defeat the purpose of our quest for robust reopening strategies. In our model, strategies are defined with respect to local conditions such as the number of cases and vaccination rates, which may be different across regions. However, after running our analysis and considering a large experimental design for a single state as we did for California, one can find a time-based reopening strategy that approximates the performance of the robust vaccination-based strategy, provided that vaccination progresses as expected. Translating a vaccination-based strategy to a time-based might prove useful for implementation purposes because vaccination strategies were defined as smooth functions of vaccination. Because vaccination rates are not equal across jurisdictions and the immunity status of the population would differ, the resulting timetable would be different for each jurisdiction, but would likely dominate alternative fixed-threshold strategies. Nevertheless, the general result that fixed-threshold reopening policies are dominated would still hold because they are a result of model structure, not parameters that describe the population of California.

Our results are generally in line with prior studies that have used similar models, but go beyond their usual findings by emphasizing which policies can be dominated. Other studies have also found a tradeoff between premature reopening amidst vaccination and COVID-19 outcomes [18–23]. Moreover, a study using an ensemble of models also found tradeoffs between the duration of interventions and COVID-19 deaths averted, with longer intervention periods being required to control the pandemic, which is in line with our findings [8]. This analysis, however, also demonstrates that strategies with fixed-case thresholds can be dominated.

The disparities exacerbated by the pandemic offer a compelling reason for more concern and rigor in defining reopening plans. While affluent populations are hedged against health and economic risks by savings and remote employment, vulnerable populations—within the US and abroad—have been experiencing the worst of the pandemic. Vulnerable populations were more likely to have had COVID-19 [40], be denied in-person education [49], and experience hunger during the pandemic [54]. How NPIs are managed in the next several months will determine the outcomes of the COVID-19 pandemic and will shape the trade-offs that these populations face. While our paper did not explicitly evaluate outcomes for distinct sub-populations within California or other jurisdictions, the evidence so far overwhelmingly points to the conclusion that populations at the margins are likely to pay a high proportion of the costs presented in this analysis, and therefore will bear the burden of dominated reopening strategies. If policymakers choose pareto-dominated reopening strategies, then it is inevitable that the already vulnerable populations will be the most affected by the consequences of dominated decisions.

Even after accounting for multiple uncertainties and simulating a wide range of strategies under many conditions, our analysis still presents limitations. While we account for three behavioral responses in our model (increase in mixing due to vaccination, change in transmissibility driven by changes in behaviors, and willingness to vaccinate), this analysis does not contain an endogenous behavioral response to changing prevalence other than the effects induced by the NPIs. In reality, people likely voluntarily react to COVID-19 prevalence, businesses adapt their operations to reduce the risk of transmission, and all these responses endogenously reduce the need for state-mandated NPIs. Similarly, when individuals or businesses relax, new surges can happen. These endogenous behavioral responses would likely introduce additional oscillations and dynamic challenges to the NPIs and might even dominate model dynamics if included. However, we are not aware of comprehensive behavioral models that we could confidently apply to our model at this stage. Better incorporating plausible behavioral mechanisms in our model is one of the next steps in our research agenda, and could reveal even more interesting results.

Although we consider immunity duration as an uncertainty, our analysis does not explicitly account for multiple components of immunological protection that will likely influence the transition to an endemic state [53]. However, doing so would likely strengthen the case for time-varying strategies. If sterilizing immunity is short-lived but second infections have a substantially lower IFR, then loss of immunity [55] will represent a smaller challenge going forward, thus requiring a lower level of intervention in the future. This analysis also does not address other long-term outcomes from alternative policies (lack of traditional education in the long-term educational outcomes, long-term COVID-19 health effects such as lung damage, mental health impacts of isolation, other illness exacerbated by reduced use of non-COVID health services, the impact of financial effects on mental and physical health, etc.).

Another set of limitations are a result of our model’s structure. The model used in this analysis is a deterministic ODE model with heterogeneous population strata, implying that eradicating COVID-19 is never achieved. While this assumption might be reasonable for US states given the lack of coordination and commitment to NPI policies, this assumption is not reasonable for smaller countries or countries with tight travel controls that were able to at least momentarily eradicate COVID-19 through swift lockdowns and prevented re-seeding for extended periods of times (e.g., New Zealand and Australia). Such countries can benefit from our framing but should adopt models that can represent eradication, which will indicate that swift lockdowns are the most effective policy, provided that there is high compliance to those measures. Because our model is defined at the state level, this analysis also does not represent interactions among different geographic levels. Accounting for multiple geographic levels and re-seeding would also likely weaken the case for constant strategies and strengthen our conclusions. Finally, this analysis does not explicitly consider distributional concerns. While these limitations have the potential to shift the tradeoff curves, they are unlikely to change our substantive results. Future iterations of this analysis might choose to include these additional mechanisms and further stress test more policies against an even wider set of uncertainties.

Despite these limitations, this analysis demonstrated that the RDM approach can be useful to stress-test a wide range of COVID-19 reopening policies under conditions of deep uncertainty. More broadly, other decision-making under deep uncertainty (DMDU) methods and tools [28] might also prove useful. While this paper only used the tools required to demonstrate that current reopening policies could be dominated, future work may make use of other available tools. Future work can explore how the use of rival framings that account for alternative objectives [56] may support different policy recommendations, as well as how Many-objective Robust Decision Making [57] could improve the trade-off surfaces we presented. Further, other many objective approaches that incorporate deep uncertainty in the many-objective search process [58, 59] may also result in alternative policies.

These approaches can accommodate the relaxation of model and methodological assumptions, allowing the policy set to be tested in an increasingly larger experimental design, helping to meet the demand for rational policy-making during a pandemic [60]. In that regard, our work contributes to a stream of analyses [61, 62] and initiatives [63] that seek to address structural uncertainties in infectious disease models. RDM and other DMDU methods share many of the goals of these approaches [62] but differ in how they addresses uncertainty. Understanding those differences and how DMDU methods can contribute to and learn from existing approaches used by infectious disease modelers might be useful to help policymakers make better decisions in this and the next pandemic.

## Supporting information

S1 Appendix Contains further details about the model, the calibration approach and the experimental design. (PDF)
