# Supplementary material for: Reopening California: Seeking robust, non-dominated COVID-19 exit strategies
Source: PLoS One. 2021 Oct 26;16(10):e0259166. doi: 10.1371/journal.pone.0259166 (PMC8547648; doi:10.1371/journal.pone.0259166)
Supplement: S1 Appendix — (PDF) [file pone.0259166.s001.pdf]

S1 Supplementary Appendix

List of Strategies

Table S1 characterizes each strategy assessed in this paper. Each row represents one strategy, their corresponding parameters, and the 75th percentile of the regret distribution over the 20,000 futures under which each strategy was tested. Lower regret represents better performance. The parameters in this table as used in the equations discussed in the Methods - Policy Levers section. Figure S1 illustrates that the information provided in table S1 is a summary of the regret distributions of each one of the strategies. While table S1 provides a summary of our findings, figure S1 illustrates how different strategies handle the challenges imposed by the uncertainties we used for our stress-tests, and how outcomes vary even when strategies are fixed. Figure S1 A shows that strategies with lower levels of caution tend to result in more deaths and lower numbers of days under NPIs. Strategies with higher levels of caution absorb those challenges by imposing longer intervention periods. Figure S1 B shows that adaptive strategies with different initial levels of caution can achieve similar results, hinting that it is possible to start with higher levels of caution and adaptively decrease the level of caution (e.g, T-12-2, T-24-2) while arriving at outcomes that are similar to outcomes obtained by strategies with constant levels of caution (e.g., C-6-1).

Strategy codes are defined as follows. The first two columns describe the characteristics of each strategy. The first letter in the strategy name represents the strategy type (C for constant caution, T for time-based, and V for vaccine-based strategies). The subsequent number represents the baseline level of caution x_b, and the third number is a sequential code to make the strategy code unique. The parameters column describes the policy levers that characterize each strategy, as described in the methods section. The final three columns present the 75th regret percentile of three metrics of interest.

Table S1. Strategy characteristics and robustness measures.

Strategy	Strategy Parameters	Deaths / 100k	NPI Days
C-0.5-1	constant	93	73
C-1.5-1	constant	50	161
C-3-1	constant	28	209
C-6-1	constant	12	238
C-12-1	constant	4	247
C-24-1	constant	0	252
V-0.5-2	$V^* = 60\%; k_c = 15$	93	72
V-1.5-2	$V^* = 60\%; k_c = 15$	53	153
V-3-2	$V^* = 60\%; k_c = 15$	31	200
V-6-2	$V^* = 60\%; k_c = 15$	14	231
V-12-2	$V^* = 60\%; k_c = 15$	5	242
V-24-2	$V^* = 60\%; k_c = 15$	1	248
V-0.5-1	$V^* = 60\%; k_c = 10$	94	71
V-1.5-1	$V^* = 60\%; k_c = 10$	54	149
V-3-1	$V^* = 60\%; k_c = 10$	32	197
V-6-1	$V^* = 60\%; k_c = 10$	15	230
V-12-1	$V^* = 60\%; k_c = 10$	5	243
V-24-1	$V^* = 60\%; k_c = 10$	1	248
V-0.5-6	$V^* = 50\%; k_c = 15$	94	70
V-1.5-6	$V^* = 50\%; k_c = 15$	58	140
V-3-6	$V^* = 50\%; k_c = 15$	37	181
V-6-6	$V^* = 50\%; k_c = 15$	19	213
V-12-6	$V^* = 50\%; k_c = 15$	8	232
V-24-6	$V^* = 50\%; k_c = 15$	3	240
V-0.5-5	$V^* = 50\%; k_c = 10$	95	68
V-1.5-5	$V^* = 50\%; k_c = 10$	59	138
V-3-5	$V^* = 50\%; k_c = 10$	37	182
V-6-5	$V^* = 50\%; k_c = 10$	19	218
V-12-5	$V^* = 50\%; k_c = 10$	8	238
V-24-5	$V^* = 50\%; k_c = 10$	2	244
V-0.5-4	$V^* = 40\%; k_c = 15$	95	65

Table S1 – continued

Strategy	Strategy Parameters	Deaths / 100k	NPI Days
V-1.5-4	$V^* = 40\%; k_c = 15$	68	123
V-3-4	$V^* = 40\%; k_c = 15$	50	154
V-6-4	$V^* = 40\%; k_c = 15$	33	177
V-12-4	$V^* = 40\%; k_c = 15$	19	202
V-24-4	$V^* = 40\%; k_c = 15$	10	222
V-0.5-3	$V^* = 40\%; k_c = 10$	97	62
V-1.5-3	$V^* = 40\%; k_c = 10$	66	124
V-3-3	$V^* = 40\%; k_c = 10$	46	161
V-6-3	$V^* = 40\%; k_c = 10$	27	195
V-12-3	$V^* = 40\%; k_c = 10$	14	224
V-24-3	$V^* = 40\%; k_c = 10$	6	238
T-0.5-6	$\alpha = 50\%; T_\alpha = 2021-09-26$	93	73
T-1.5-6	$\alpha = 50\%; T_\alpha = 2021-09-26$	53	155
T-3-6	$\alpha = 50\%; T_\alpha = 2021-09-26$	31	200
T-6-6	$\alpha = 50\%; T_\alpha = 2021-09-26$	14	223
T-12-6	$\alpha = 50\%; T_\alpha = 2021-09-26$	5	238
T-24-6	$\alpha = 50\%; T_\alpha = 2021-09-26$	1	249
T-0.5-5	$\alpha = 50\%; T_\alpha = 2021-07-04$	93	72
T-1.5-5	$\alpha = 50\%; T_\alpha = 2021-07-04$	56	140
T-3-5	$\alpha = 50\%; T_\alpha = 2021-07-04$	34	191
T-6-5	$\alpha = 50\%; T_\alpha = 2021-07-04$	16	224
T-12-5	$\alpha = 50\%; T_\alpha = 2021-07-04$	6	240
T-24-5	$\alpha = 50\%; T_\alpha = 2021-07-04$	1	248
T-0.5-4	$\alpha = 50\%; T_\alpha = 2021-03-10$	104	53
T-1.5-4	$\alpha = 50\%; T_\alpha = 2021-03-10$	71	127
T-3-4	$\alpha = 50\%; T_\alpha = 2021-03-10$	46	165
T-6-4	$\alpha = 50\%; T_\alpha = 2021-03-10$	24	211
T-12-4	$\alpha = 50\%; T_\alpha = 2021-03-10$	11	222
T-24-4	$\alpha = 50\%; T_\alpha = 2021-03-10$	4	229
T-0.5-3	$\alpha = 10\%; T_\alpha = 2021-09-26$	93	73
T-1.5-3	$\alpha = 10\%; T_\alpha = 2021-09-26$	53	149
T-3-3	$\alpha = 10\%; T_\alpha = 2021-09-26$	36	182
T-6-3	$\alpha = 10\%; T_\alpha = 2021-09-26$	22	204
T-12-3	$\alpha = 10\%; T_\alpha = 2021-09-26$	12	217
T-24-3	$\alpha = 10\%; T_\alpha = 2021-09-26$	5	225
T-0.5-2	$\alpha = 10\%; T_\alpha = 2021-07-04$	93	72
T-1.5-2	$\alpha = 10\%; T_\alpha = 2021-07-04$	64	130
T-3-2	$\alpha = 10\%; T_\alpha = 2021-07-04$	54	147
T-6-2	$\alpha = 10\%; T_\alpha = 2021-07-04$	33	168
T-12-2	$\alpha = 10\%; T_\alpha = 2021-07-04$	18	192
T-24-2	$\alpha = 10\%; T_\alpha = 2021-07-04$	9	213
T-0.5-1	$\alpha = 10\%; T_\alpha = 2021-03-10$	124	0
T-1.5-1	$\alpha = 10\%; T_\alpha = 2021-03-10$	109	65
T-3-1	$\alpha = 10\%; T_\alpha = 2021-03-10$	93	92
T-6-1	$\alpha = 10\%; T_\alpha = 2021-03-10$	71	112
T-12-1	$\alpha = 10\%; T_\alpha = 2021-03-10$	47	130
T-24-1	$\alpha = 10\%; T_\alpha = 2021-03-10$	26	161

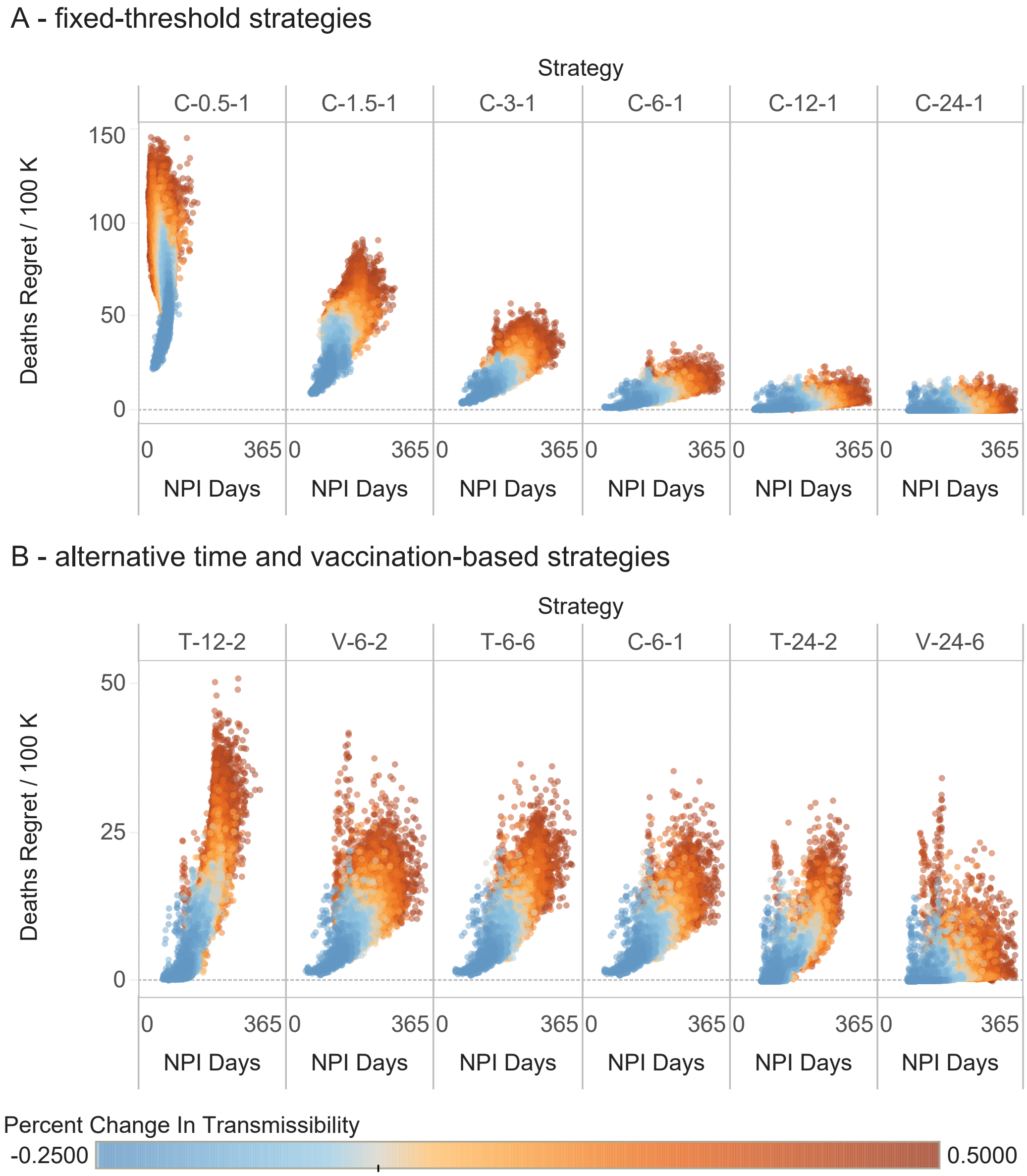

Fig. S1. Regret distributions for a sub-set of strategies. This figure presents the performance of a subset of the strategies presented in terms of Deaths / 100 k people regret and Number of Days under NPIs regret. Each dot in this plot represents the performance of each strategy in one of the 20,000 futures. The color gradient represents Change in Transmissibility uncertainty.

Model

The model used in this analysis is based on our previously published ODE model (1) and its recent extension (2). Figure S2 presents an overview of our compartmental model, whereby individuals in our population progress over the different stages of the infection.

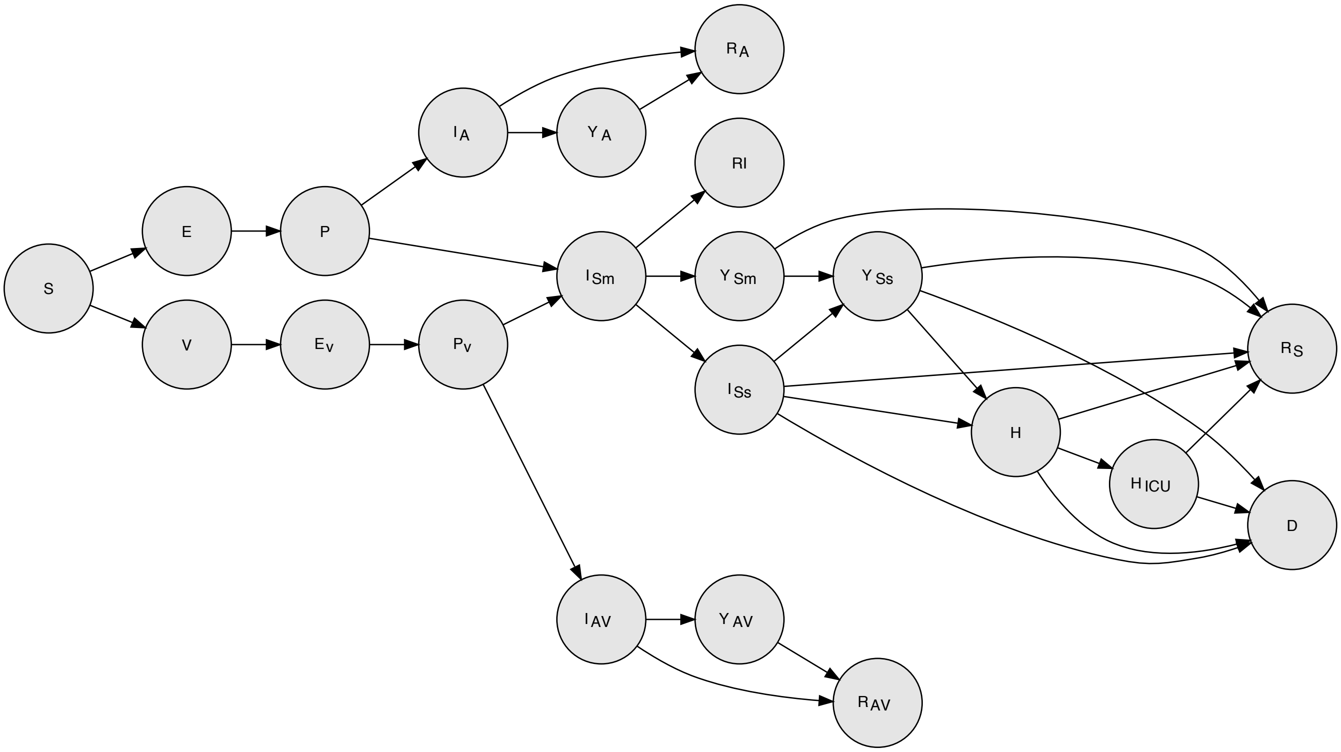

Fig. S2. Compartmental Model Structure. Each circle in this figure represents a progression state, each subdivided in 5 population strata. For simplicity and clarity, the figure omits arrows representing loss of immunity (from the recovered states to the S state). See equations for details.

Individuals in our population are divided into 21 compartments. The set of compartments that are common to our NPI-only model include: The noninfected and susceptible (S), the exposed and infected but not yet infectious (E), the presymptomatic or primary infectious stage (P), the infected with mild symptoms (I_{Sm}), the infected with severe symptoms (I_{Ss}), the diagnosed infected with mild symptoms (Y_{Sm}), the diagnosed infected with severe symptoms (Y_{Ss}), the non-ICU hospitalized (H), the hospitalized in the ICU (H_{ICU}), the infected asymptomatic (I_A), the diagnosed infected asymptomatic (Y_A), and those that died (D). We assume that individuals in the P and I_A compartments are completely asymptomatic and thus are unaware of being infectious. All those compartments were present in our previous work (1, 2). As in our previous model, this model does contain multiple population strata, but it does not contain geographic patches within a US state. Although this may seem an important limitation, the COVID-19 case data in California suggests that COVID-19 waves occurred at the same time across counties. Because reopening policies are defined at the state level, and COVID-19 waves do not seem to respect county boundaries within states, we believe our model is defined at an appropriate geographic level given the questions posed in the problem framing section.

The model used in this paper includes new compartments aiming to represent vaccination roll-out. Here, we focus our description on these compartments. New compartments include those who have received a full vaccination dose (V), the vaccinated who have been infected and are in the exposed and infected but not yet infectious stage (E_v), the vaccinated in the presymptomatic infectious stage (P_v), the vaccinated in the infected asymptomatic (I_{Av}) and those diagnosed infected asymptomatic (Y_{Av}). This model also has three distinct recovered stages R_I, R_A and R_{Av} allowing us to respectively track those that have recovered having been symptomatic, non-vaccinated asymptomatic, and vaccinated asymptomatic.

The arrows connecting the disease states describe the progression rates between the different compartments. We assume that mild symptoms are a dry cough and a fever, while severe symptoms also include shortness of breath. The sum of the population in all of the states gives the total population N. However, we assume that $N = 1$ and

thus each state variable gives the proportion of the population belonging to that state.

Model Formulation. Our compartmental model is described by the following set of coupled ordinary differential equations (ODEs):

$$
\begin{aligned}
\dot{S} &= -\lambda S - \omega(t)S, && [1]\\
\dot{V} &= \omega S - \lambda_v S, && [2]\\
\dot{E} &= \lambda S - \nu E, && [3]\\
\dot{P} &= \nu E - (\gamma_S + \gamma_A)P, && [4]\\
\dot{E}_v &= \lambda_v V - \nu_v E_v, && [5]\\
\dot{P}_v &= \nu_v E_v - (\gamma_V + \gamma_{Av})P, && [6]\\
\dot{I}_A &= \gamma_A P - [\xi_A + \zeta_A(t)]I_A, && [7]\\
\dot{Y}_A &= \zeta_A(t)I_A - \xi_A^* Y_A, && [8]\\
\dot{I}_{Av} &= \gamma_{Av} P - [\xi_A + \zeta_{Av}(t)]I_{Av}, && [9]\\
\dot{Y}_{Av} &= \zeta_{Av}(t)I_{Av} - \xi_A^* Y_{Av}, && [10]\\
\dot{I}_{Sm} &= \gamma_S P - [\upsilon + \xi_m + \zeta_S(t)]I_{Sm}, && [11]\\
\dot{Y}_{Sm} &= \zeta_S(t)I_{Sm} - [\upsilon^* + \xi_m^*]Y_{Sm}, && [12]\\
\dot{I}_{Ss} &= \upsilon I_{Sm} - [\xi_s + \mu_s + hA_H + (1 - A_H)\zeta_S(t)]I_{Ss}, && [13]\\
\dot{Y}_{Ss} &= (1 - A_H)\zeta_S(t)I_{Ss} + \upsilon^* Y_{Sm} - [\xi_s^* + \mu_s + h^* A_H]Y_{Ss}, && [14]\\
\dot{H} &= A[hI_{Ss} + h^* Y_{Ss}] - [\mu_H + \chi + \xi_H]H, && [15]\\
\dot{H}_{ICU} &= A_{ICU}\chi H - [\mu_{ICU} + \xi_{ICU}]H_{ICU}, && [16]\\
\dot{R}_A &= \xi_A I_A + \xi_A^* Y_A, && [17]\\
\dot{R}_{Av} &= \xi_A I_{Av} + \xi_A^* Y_{Av}, && [18]\\
\dot{R}_S &= \xi_m I_{Sm} + \xi_m^* Y_{Sm} + \xi_s I_{Ss} + \xi_s^* Y_{Ss} + \xi_H H + \xi_{ICU} H_{ICU}, && [19]\\
\dot{D} &= \mu_S(I_{Ss} + Y_{Ss}) + [\mu_H + (1 - A_{ICU})\chi]H + \mu_{ICU} H_{ICU}. && [20]
\end{aligned}
$$

Many of the ODEs and transition rates in equations 1 -20 are the same as those used by our first COVID-19 transmission model described in our recent work (2). Here we focus on providing a high-level overview of the way NPIs are represented in our model and on describing the additions made to the ODEs and the model. Vaccination is the most important addition compared to our previous model, and most of the description is centered around how we model vaccination.

The additional disease compartments include new disease-specific progression rates. In particular, disease progression rates for those that have vaccinate can differ from those who have not. The vaccination rate is given by the parameter $\omega(t)$, described later in this paper. We denote the per-person progression rate from exposure to the presymptomatic for those vaccinated by ν_v. The progression rates for those who vaccinate differs from the progression rates for those who do not vaccinate. Hence, the progression rates γ_v and γ_{Av} are not respectively equal to γ_S and γ_A. However, we assume that the overall duration of the presymptomatic phase does not change with vaccination. Hence, $(\gamma_v + \gamma_{Av})^{-1} = (\gamma_S + \gamma_A)^{-1}$. However, the proportion a_v of those vaccinated that remain asymptomatic is higher than the same proportion a_0 of those who did not. Hence, $a_v > a_0$, where $a_v = \gamma_{Av}(\gamma_v + \gamma_{Av})^{-1}$ and $a_0 = \gamma_A(\gamma_S + \gamma_A)^{-1}$. We assume that those who have been vaccinated but get infected and develop mild symptoms progress through the disease's clinical states as if they were not vaccinated. Hence, for these people, we assume that the vaccine has failed and no longer provides benefits. However, the majority of vaccinated individuals will continue to stay asymptomatic. Their disease progression rate is the same as those asymptomatic who did not vaccinate except for the detection rate. We assume that those who are vaccinated and asymptomatic have a lower rate of seeking to get tested, and hence $\zeta_{Av} < \zeta_A$.

Our model also tracks additional outputs. We compute the true cumulative case counts $\dot{C}_T$; the reported cumulative case counts $\dot{C}_R$, the cumulative number of people tests $\dot{T}$, the reported recovered $\dot{R}_R$, the reported deaths $\dot{D}_R$ and the reported case-fatality rate $\text{CFR}_R(t)$. These output quantities are respectively computed using the following equations.

$$\dot{C}_T = \lambda, \quad [21]$$

$$\dot{C}_R = \zeta_S[I_{Sm} + (1 - A_H)I_{Ss}] + hA_H I_{Ss} + \zeta_A I_A, \quad [22]$$

$$\dot{T} = \dot{C}_R + \zeta(S + E + P), \quad [23]$$

$$\dot{R}_R = \xi_A^* Y_A + \xi_m^* Y_{Sm} + \xi_s^* Y_{Ss} + \xi_H H + + \xi_{ICU} H_{ICU}, \quad [24]$$

$$\dot{D}_R = \dot{D} - \mu_S I_{Ss}, \quad [25]$$

$$\text{CFR}_R(t) = D_R(t)/C_R(t). \quad [26]$$

Population Groups and Mixing. Our model considers different population groups or strata. We consider five population strata, including the front-line essential workers (FLEW), the employed non-FLEW, the unemployed, minors of age below 17, and seniors of 65 and above. The first three population strata only include those aged 18 to 64. The prognosis parameters that enter the ODEs depend on the population strata. Prognosis parameters include the proportion of people that develop symptoms (i.e., γ_S, γ_A, γ_V and γ_{Av}), the proportion of symptomatic who develop severe and critical symptoms (i.e., υ and χ), and the proportion of critical cases that lead to death without pharmaceutical treatment (i.e., μ_{ICU}).

The structure of the model is expressed as an array of ODEs, where the disease progression dynamics for each stratum are expressed by equations 1-20. This formulation extends the model from the more conventional version of a single-strata compartment model that assumes homogeneous mixing and implicit interactions within the population. Heterogeneity in disease transmission is introduced by strata-dependent mixing contact rates describing the variations in how people belonging to the different population strata mix with each other. These strata-dependent mixing contact rates control the transmission dynamics, specifically the *force of infection* terms λ and λ_v that enter the ODEs.

We consider six different mixing modes including household, work, school, commercial, recreation, and other. We used a combination of a network-based dataset and self-reported survey data to create matrices describing the average daily contacts between each stratum in each mixing mode (3, 4). We decompose these matrices into a set of row normalized five-by-five mixing matrices $\mathbf{M}_m$, column normalized contact vectors κ_m, and scalar mode weight w_m for each mixing mode labeled by the index m. The total contact matrix, K is calculated by a weighted sum of the mode-specific contact matrices $\mathbf{K}_m$, and expressed as

$$\mathbf{K} = \sum_m w_m[\kappa_m \odot \mathbf{M}_m] = \sum_m w_m \mathbf{K}_m, \quad [27]$$

where $\odot$ denotes the element-wise multiplication. The weights, w_m give the proportion of contacts (or duration of contacts) of how people mix over the different mixing modes. Under the disease-free status-quo conditions these weights sum to one, hence $\sum_m w_m = 1$.

Modeling SARS-CoV-2 transmission. SARS-CoV-2 transmission is modeled by the force of infection λ which characterizes how infectious people in each disease state infect others. We express the force of infection as a vector of five elements, one for each stratum and expressed as

$$\lambda(t) = k_\lambda c_{eff}\beta_{eff}\mathbf{K} \cdot \sum_{X_I} m_{X_I}\mathbf{X}_I(t), \quad [28]$$

where c_{eff} represents the effective contact rate, and β_{eff} effective transmissibility, and X_I represents the set of the infectious disease compartments. This set includes all the disease stages that are infectious, including those that follow from disease transmission of vaccinated people, namely P_v, I_{Av} and Y_{Av}. People who have COVID-19 symptoms or are diagnosed are less likely to mix socially. Moreover, people in the early stages of the disease are more infectious. Hence, we use the coefficients m_{X_I} to represents the multiplicative reduction factor for infectious states X_I that scale the transmission rate relative to the asymptomatic and unaware of being infectious. We compute the value of the product $c_{eff}\beta_{eff}$ by setting the values of the basic reproductive ratio R_0. By using the next-generation matrix method we find that $c_{eff}\beta_{eff} = R_0/t_{eff}$, where the time scale t_{eff} is expressed in terms of the multiplicative coefficients m_{X_I} and the values of the disease progression rates (2, 5). The multiplicative factor k_λ represents a calibration constant. Heterogeneity in transmission rates across the population strata is accomplished by the total contact matrix K.

Our model assumes imperfect vaccines whereby those who vaccinate may still contract the disease and become infectious and symptomatic. In section we describe how we model the efficacy of the vaccine and the virus transmission amongst those who vaccinated.

Nonpharmaceutical Intervention Levels. Nonpharmaceutical interventions (NPIs) based on social distancing reduce the total number of unique contacts. They are modeled using a different set of scalar weights w_m that enter equation 27, and are such that their sum is less than one. For example, we can set all values of w_m to zero except for $m =$ Household mixing, which retains its original value or perhaps increases it. Additionally, we can modify the mixing matrix $\mathbf{M}_m$ for $m =$ Household mixing to describing a different behavior of age group mixing within a household due to the new social-distancing measures. Hence, we obtain a different contact matrix K. Specifically, to model the impact of reduced mixing from NPI level n on mode m, we define a diagonal matrix $\mathbf{\Phi}_m^{\{n\}}$. The diagonal elements of $\mathbf{\Phi}_m^{\{n\}}$ specify the reduction in mixing for each stratum in mode m relative to the disease-free state. For interventions that apply to all strata (i.e., where each stratum changes their mixing by the same proportion), such as the closure of schools, all diagonal elements of $\mathbf{\Phi}_m^{\{n\}}$ have the same value. However, there are some interventions that only apply to some strata and not others. For example, the case when only essential front-line workers are expected to attend their workplaces. In such cases, the diagonal elements of $\mathbf{\Phi}_m^{\{n\}}$ take on different values, each specifying the strata-mode specific impact of the NPI. Hence, the expression for $\mathbf{K}^{\{n\}}$ that accounts for the impact of NPIs is:

$$\mathbf{K}^{\{n\}} = \sum_m w_m \left\{ (\mathbf{\Phi}_m^{\{n\}})^{\frac{1}{2}} \mathbf{K}_m (\mathbf{\Phi}_m^{\{n\}})^{\frac{1}{2}} \right\}. \qquad [29]$$

Table S2 provides a description of the intervention levels which are denoted by the index n. These intervention levels are used in our model to mechanistically change the transmission processes at different mixing modes and the goal of the model is to compute the consequences of that level of transmission on health outcomes. However, using only those outcomes is not sufficient to properly inform decision-making. As discussed in the methods section, it is desirable to use additional outcome measures to evaluate the pareto-efficiency of alternative strategies. Because minorities and workers at high-contact service industries (e.g., hospitality and leisure) are more likely to face unemployment and income loss during the pandemic (6), accounting for the effects of policies on those populations is essential if modelers seek to provide comprehensive decision support to policymakers. For these reasons, we seek to use measures that are monotonically increasing relative to the unknown marginal effect of NPIs on social welfare. This paper uses the number of days of NPIs as the primary measure. In addition to that measure we obtain an estimate of the weekly income loss incurred in each of the NPI levels using the baseline estimates from the general equilibrium economic model (7). At the end of the simulation run, we aggregate the income loss incurred under each NPI level.

Table S2. Nonpharmaceutical intervention levels.

NPI Level (n)	Description
Level 1: No Intervention	No Intervention
Level 2: Close schools	All schools are closed.
Level 3: Close schools, bars, and restaurants; and ban large events	In addition to school closures, all bars' and restaurants' dine-in services are closed, only allowing for take-out options. Also, large gatherings are banned.
Level 4: Close schools, bars, and restaurants; ban large events; and close nonessential businesses	In addition to school, bar, and restaurant closures, all nonessential businesses are closed.
Level 5: Close schools, bars, and restaurants; ban large events; close nonessential businesses; and shelter in place for the most vulnerable	In addition to the closure of all nonessential businesses, a shelter in place recommended for the vulnerable population, including the elderly, children, and other at-risk populations.
Level 6: Close schools, bars, and restaurants; ban large events; close nonessential businesses; and shelter in place for everyone but essential workers	In addition to the interventions above, shelter in place order is issued for everyone but essential workers.

Modeling Adaptive Strategies. This paper presented only three alternative types of adaptive strategies, which resulted in 78 alternative strategies. Yet, there are many potential ways to frame and model reopening policies. Instead of addressing the question of when society can reopen schools by simulating outcomes under a simple set of rules with an exogenous NPI time-series, we use an endogenous controller to represent a strategy and ask how policymakers should manage their level of caution over time. The difference between the two questions is important. While other studies (8) and our early work (1) addressed the impact of specific fixed policies, this approach does not address the

important question of how to adapt policies over time conditional on vaccination. While the first question allows a simple comparison and is more intuitive, the first framing inevitably results in large outbreaks if stringent policies are not followed, and might lead to recommendations that are vulnerable to new strains with higher transmissibility. Because the benefits of NPIs are a non-linear function of the immunity status in the population, and because immunity is changing over time, a set of fixed intervention schedules can result in a menu of options that would be pareto-dominated if a wider set of options was included. This is the main concern and motivation for expanding the option set with alternative strategies.

Framing policies as endogenous also has disadvantages. This formulation implies that policymakers can and will sustain a coherent level of caution over time, and strictly follow that strategy. We remedy this disadvantage by conceptualizing the level of caution as a potentially time-varying control and implementing a stopping condition to cease the use of NPIs once an immunity threshold based on vaccination is crossed. This approach allows us to answer specific questions such as "when interventions can be lifted" while using an endogenous controller that is more robust to uncertainties and representative of adaptive policies. Rather than being an input, the date when NPIs are relaxed is an outcome - a function of policy levers and the uncertainties. The rationale behind this formulation is that policymakers will face higher pressure to relax policies as a wider proportion of the population is vaccinated.

One approach to reconciling the two approaches could be to run the analysis using the endogenous policies over a wide range of futures and then derive an NPI time-series from strategies that were not pareto-dominated. This policy could be translated to an exogenous policy. We did not explicitly do that in this paper because using that NPI time-series for other states or countries could be potentially misleading. However, that approach could be potentially useful for public health departments that wish to translate dynamic, endogenous policies to more interpretable prescriptions.

Vaccination. Our model accounts for a phased vaccination rollout, where a one-dose or a two-dose vaccine is distributed to population strata in order of priority.In our model, those who are immunized (either with a two-dose or a one-dose vaccine) enter the vaccinated compartment V. Our model represents vaccination supply and demand separately.

Vaccination capacity is the average rate at which vaccine courses (VCs) can be administered by state. Vaccination capacity increases over time. We assume that, starting from the day when vaccines start to be administered, denoted by t_v, the daily supply rate of VCs $s_v(t)$ increases from zero to a maximum daily rate $s_v^{\{\max\}}$ based on the sigmoid function

$$s_v(t) = s_v^{\{\max\}} \frac{e^{\ln 2 \cdot (t-t_v)/\tau_v} - 1}{e^{\ln 2 \cdot (t-t_v)/\tau_v}} \text{ for } t \geq t_v. \quad [30]$$

τ_v is the time scale of capacity increase such that $s_v(\tau_v) = s_v^{\{\max\}}/2$. We denote the daily number of VCs utilized in each stratum by the vector $\mathbf{u}(t)$, and the total number of available VCs as a stock variable $v(t)$. The change in the daily number of available VCs is equal to the difference between the daily number of VCs supplied $s_v(t)$, and the sum of the daily number of VCs utilized across the population strata, which we denote by $u(t)$. The latter is equal to the sum of the elements of the vector $\mathbf{u}(t)$. Our model tracks the total number of available VCs $v(t)$ by treating it as a stock using the following ODE

$$\dot{v}(t) = s_v(t) - u(t). \quad [31]$$

Our model tracks the total number of utilized VCs in each stratum. This is denoted by the vector $\mathbf{U}(t)$ and is given by the time integral of $\mathbf{u}(t)$. The daily number of VCs used $\mathbf{u}(t)$ depends on the vaccination allocation policy and demand. At the start of the vaccine rollout, we assume that policymakers specify a vaccination allocation policy. The policy is denoted by a vector $\mathbf{A}_V^*$. Its elements determine the proportion of vaccines allocated to each population strata, and hence they sum to one. The vector $\mathbf{A}_V^*$ specifies the initial allocation policy, such that higher priority groups have higher values. It is constant over time. However, the actual allocation policy, denoted by $\mathbf{A}_V(t)$ changes over time because willing members of priority groups deplete as vaccines are distributed. $\mathbf{A}_V(t)$ depends on the proportion of each stratum willing and eligible to receive additional vaccine doses and the vaccine allocation $\mathbf{A}_V^*$.

We define the vector $\mathbf{W}$ as the proportion of each stratum approved to receive the vaccine and willing to get vaccinated. We then construct an indicator function, $\mathcal{I}_V(t)$, which describes if there is still demand in each stratum at time t. This allows us to 'switch off' vaccine supply to strata that have been fully vaccinated. The indicator function $\mathcal{I}_V(t)$ is expressed as a Heaviside step function $\mathcal{H}(x)$, and compares the number of willing and eligible individuals, $\mathbf{W}$, to the total number of utilized VCs, $\mathbf{U}(t)$, element by element:

$$\mathcal{I}_V(t) = \mathcal{H}[\mathbf{W} - \mathbf{U}(t)]. \quad [32]$$

Each element of $\mathcal{I}_V(t)$ represents a population stratum. The value of the element is equal to 1 as long as there are still individuals in the stratum approved and willing to vaccinate and equal to 0 otherwise. As of January 2021, the FDA

has approved the vaccines for everyone except minors of less than 16. Hence, the value of the $\mathcal{I}_V(t)$ for the youngest population strata only considers whether all eligible minors have received the vaccine. The normalized element-wise multiplication of vectors $\mathbf{A}_V^*$ and $\mathbf{D}_V(t)$, gives the time-varying allocation vector $\mathbf{A}_V(t)$, and is expressed as

$$\mathbf{A}_V(t) = \mathcal{N}\left[\mathbf{A}_V^* \odot \mathcal{I}_V(t)\right]. \quad [33]$$

The function $\mathcal{N}(.)$ is a normalization function such that the sum of the elements of $\mathbf{A}_V(t)$ is equal to one. Thus, as the highest priority stratum has all willing members vaccinated, this value in $\mathbf{A}_V(t)$ is set to zero, and the priority on other strata are increased.

At the beginning of the rollout, we expect the demand for vaccines to be high. For this case, supply will be limited, and the daily rate of vaccinations in each stratum is given by $s_v(t) \cdot \mathbf{A}_V(t)$. However, when the vaccination capacity is no longer a constraint, the daily rate of vaccinations in each stratum no longer depends on the initial vaccination policy $\mathbf{A}_V^*$. Instead, it depends on demand, which we denote as $\mathbf{D}_V(t)$. As mentioned, our indicator function $\mathcal{I}_V(t)$ signals whether demand is present for each stratum. We assume that demand is limited to the unvaccinated susceptible (S), and recovered (R_A and R_S) population. Hence, the vector representing the demand for VCs in each population strata is given by an element-wise multiplication of vectors $S + R_A + R_S$, and $\mathcal{I}_V(t)$, and expressed as

$$\mathbf{D}_V(t) = (\mathbf{S} + \mathbf{R_A} + \mathbf{R_S}) \odot \mathcal{I}_V(t). \quad [34]$$

When people no longer perceive the vaccination capacity as constrained, they may seek to get vaccinated at a different rate, $s_w(t)$. We assume that this probability is the same across the population strata and does not vary with time. We also make the assumption that vaccination rate is independent of the Nonpharmaceutical intervention policy. Hence, in our model the daily consumption rate of VCs is the minimum of supply and demand in each strata:

$$u(t) = \mathcal{P}_{\min}\left[s_v(t) \cdot \mathbf{A}_V(t), s_w \cdot \mathbf{D}_V(t)\right] \quad [35]$$

The function $\mathcal{P}_{\min}[\mathbf{x}, \mathbf{y}]$ is the parallel minimum and returns the element-wise minimum between the vectors $\mathbf{x}$ and $\mathbf{y}$. We can convert this into a per-person daily consumption rate of VCs among the susceptible:

$$\omega(t) = \frac{u(t)}{\mathbf{S} + \mathbf{R_S} + \mathbf{R_A}} \quad [36]$$

Both the Heaviside step function and the parallel minimum introduce abrupt changes in the model dynamics and lead to a significant increase in stiffness of the ODEs. This is problematic because it significantly slows the numerical solvers. To resolve this issue, we used a continuous approximation to these functions. For example, we approximated the step function with a very steep sigmoid function.

Vaccination Efficacy. Our model separately considers the vaccine efficacy in protecting from disease transmission and in preventing symptoms. As inputs, the model requires the specification of the vaccine's overall efficacy of e_v and efficacy in protecting from disease transmission e_{tv}. The overall efficacy is given by

$$e_v = 1 - F_v/F_0, \quad [37]$$

where F_v is the proportion of individuals in the treatment group that during phase 3 vaccine trials reported having symptoms, and F_0 represents the same proportion in the control-placebo group. We can express F_v and F_0 in terms of the e_v as

$$F_v = \beta(1 - e_{tv})(1 - a_v) \quad [38]$$
$$F_0 = \beta \cdot (1 - a_0) \quad [39]$$

where β is the overall transmissibility common to the treatment and the control group. The proportions a_v and a_0 respectively represent the probabilities of remaining asymptomatic after being infected for those who do and do not vaccinate. It follows that we can express a_v in terms of a_0 using the vaccine's efficacy values by the expression

$$a_v = 1 - \frac{(1 - e_v)(1 - a)}{1 - e_{tv}}. \quad [40]$$

Therefore, by specifying a, e_v and e_{tv} we can find the value for a_v and hence the relative probabilities that those who are vaccinated develop mild or severe disease, expressed through γ_V and γ_{Av}.

To model the transmission of SARS-CoV-2 to those who have vaccinated we consider both the efficacy of the vaccine in protecting from disease transmission e_{tv} as well as the increase in the rate of social mixing of those who have vaccinated. Following from section , we express the force of infection on those who have vaccinated as

$$\lambda_v(t) = k_\lambda c_{eff} \beta_{eff} (1 - e_{tv}) m_v \mathbf{K} \cdot \sum_{X_I} m_{X_I} \mathbf{X}_I(t). \quad [41]$$

The coefficient m_v represents a multiplicative factor that accounts for the overall effect of behavior changes of the people who vaccinate. For example, these behavioral changes include the tendency for those who vaccinate to be less willing to comply with NPIs and continue to wear their masks, and to generally increase their social mixing rate. In the equations 28 and 41, X_I represents the set of all infectious states and it includes the states P_v, I_{Av} and Y_{Av}. These three infectious states follow from the disease transmission to those who have vaccinated. Hence, both equations depend on the coefficients m_{P_v}, $m_{I_{Av}}$ and $m_{Y_{Av}}$ that scale the transmission rate relative to the asymptomatic and unaware of being infectious. The multiplicative factor m_v is included as part of these three coefficients. For example, we set $m_{P_v} = m_v m_P$, and likewise for the other two factors. Hence, equation 41 considers a squared behavioral effect whereby people who are vaccinated increasingly mix amongst each other by an overall multiplicative factor of m_v^2.

Additional Mechanisms. Our model includes additional mechanisms that influence the long-term transmission dynamics. These include seasonality and loss of immunity. We model seasonality in transmission by multiplying $c_{eff}\beta_{eff}$ by a time-varying term denoted by $\vartheta(t)$ that has an average value equal to one over a year. We use a sinusoidal function to describe $\vartheta(t)$. A parameter s controls for the strength of the seasonal effect in the time-varying function $\vartheta(t)$. To model loss of immunity (9), we allow those who recover can become susceptible again. We use a first-order boxcar method and include an intermediate recovered compartment R_B, which recovered people transition into before losing immunity and returning the susceptible population pool S. These mechanisms are described in more detail in our prior work (2).

Calibration

Model calibration was performed using the Incremental Mixture Approximate Bayesian Computation (IMABC) algorithm (10). The calibration approach requires the specification of parameter priors $\pi(\theta_c)$ and calibration targets y^*. IMABC begins with a rejection-based approximate Bayesian computation (ABC) step, drawing a sample of parameters θ_c of size N_0 from their prior distribution $\pi(\theta_c)$, simulating calibration targets y_t, and accepting parameters that yield simulated outcomes near observed targets within initial tolerance bounds of $y_t^* \pm \epsilon_{f,t}$. Next, the sample is iteratively updated by drawing additional candidate parameters from a mixture of multivariate normal distributions, centered at the parameters that yield simulated targets that are closest to observed targets. IMABC uses $(y^* - y_t)^2/y_t^2$ as a distance metric of simulated outcomes from the observed data. As more points are accepted, the initial tolerance bounds are narrowed, and parameters that yield simulated targets outside of these new bounds are removed. The algorithm has converged when it obtains the requested number of draws that are within final tolerance intervals, $y_t^* \pm \epsilon_{f,t}$. Once the algorithm has converged, posterior estimates can be obtained using a weighted sample from accepted parameter vectors (10). The weights account for the selection of the sample, using the normal mixture distributions.

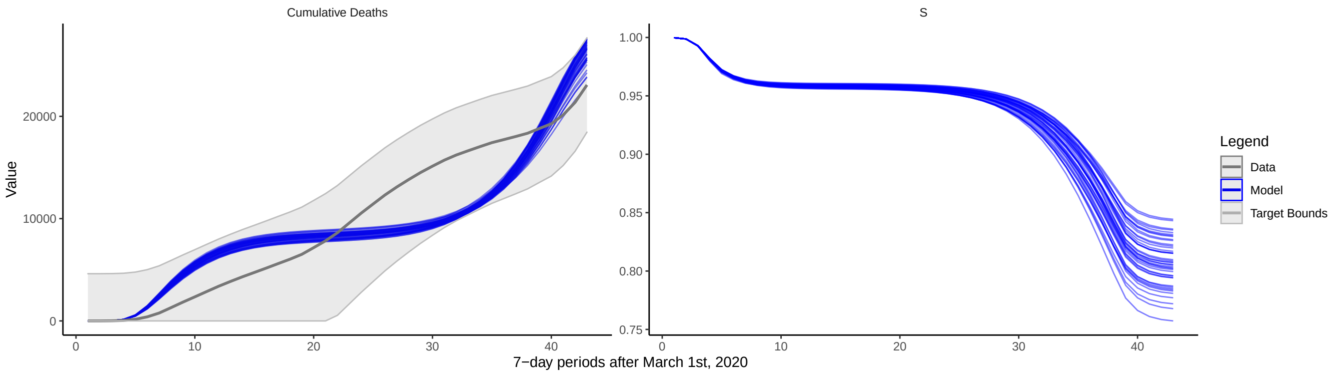

(a) Data collapsed at 7-day time periods

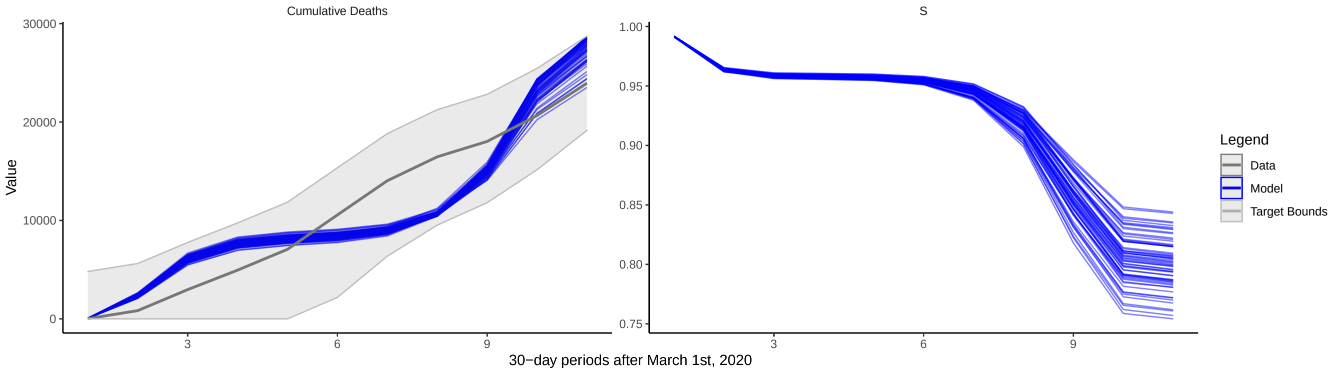

(b) Data collapsed at 30-day time periods

Fig. S3. Calibration results for California. The blue lines are the outputs of the calibrated model across three metrics of interest. The data and the calibration target bounds are displayed in gray.

We calibrate the model using COVID-19 deaths timeseries from March 1st, 2020 through December 25th, 2020. We use reported COVID-19 deaths because that is the most reliable indicator available and the main outcome of interest for this analysis. This time-series does not include all-cause excess mortality, which is beyond the scope of our model. We use this time period because we are interested in how policymakers should shift their reopening strategy after vaccination started in the US. We use cumulative deaths time-series at the state level, collapsed at ten 30-day intervals $y_t, t \in \{1, ..., 10\}$ starting in March 1st, 2020 through December 25th, 2020. Figure S3(b) shows the data we use and illustrates the model runs that the algorithm selects. We set the initial tolerance level as $\epsilon_i = max(y_t)$ and the final tolerance level as $\epsilon_f = 0.2max(y_t)$ where y_t is the cumulative number of deaths. Therefore, the goal of the algorithm is to find model runs that are within the envelope $y_t \pm \epsilon_{f,t}$ of the cumulative death time series illustrated in the graph. In addition to the death time series calibration target, we also require the number of susceptible individuals in the model to be greater than 65%, seeking to find model runs that are consistent with seroprevalence data.

We choose to use 30-day time periods with the aim of reducing the number of individual targets that the calibration procedure needs to track. As figure S3(a) illustrates, choosing another time period (e.g, a 7-day) would yield similar results. Figure S3 also illustrates that the model does not fit the surge in cases around month number 6 in California (September 2020). That is the case because our model does not contain time-varying mixing parameters that could absorb that surge. For the purposes of this analysis, we argue that adding more parameters to the model calibration phase could be problematic*.

The calibration results presented above are a function of our model structure presented earlier, the calibration targets and their tolerance interval used, and a set of parameters. Parameters used during the calibration run are divided into three sets: calibrated parameters (C), parameters that were fixed during calibration (F), and parameters that were fixed during calibration but later explored as deep uncertainties (F, D) using a new experimental design. Table S3 presents each parameter, the set to which they belong, a formula or symbol relating the parameter to our

* Adding time-varying parameters to the model seeking to improve model fit without a mechanistic explanation for the summer surge can be problematic for the purposes of this analysis. For example, the surge could signal an increase in pandemic fatigue and not a month-level seasonal phenomenon. Our future work might explore better ways of incorporating realistic behavioral components in our model that could better explain the unexplained surges using more calibration targets to inform the model. For the purposes of this analysis, we chose not to overfit the model to the data and preserve only parameters that represent known mechanisms.

model and related sources. The value column includes the parameter mode and minimum and maximum bounds used during calibration.

Table S3. Model Parameters.

Set	Parameter	Formula or Symbol	Value	Sources
C	Proportion of infections which are asymptomatic	$\frac{\gamma_A}{\gamma_A+\gamma_S}$	0.25 [0.15, 0.5]	(11–14)
C	Proportion of symptomatic infections which are severe (require hospitalization)	$\frac{\upsilon}{\upsilon+\xi_m+\mu_S}$	0.05 [0.03, 0.07]	(2)
C	Proportion of severe cases which are critical (require ICU admission)	$\frac{\chi}{\chi+\xi_H+\mu_H}$	0.32 [0.26, 0.38]	(14, 15)
C	Initial proportion of critical cases which result in death	$\frac{\mu_{ICU}}{\xi_{ICU}+\mu_{ICU}}$	0.75 [0.7, 0.8]	(15–17)
C	Magnitude of seasonal effect when it is at its peak	s	0.2 [0.15, 0.3]	(2)
C	Intervention Calibration Factor	θ	2 [0.5, 3.5]	(2)
C	Magnitude of behavioral adaptation factor after lockdown.	b_h	0.45 [0.1, 0.8]	(2)
C	Reduction in ICU death proportion when maximum treatment efficacy is achieved	N/A	0.5 [0.4, 0.6]	(16)
C	1/ rate at which treatment improves (months)	N/A	6 [3, 9]	(16)
C	Increased progression rate for tested individuals	κ_ξ	2 [1.33, 4]	(2)
F	Infectivity of mild symptomatic stage relative to asymptomatic infectivity.	m_{Im}	0.83	(14, 18–20)
F	Infectivity of severe symptomatic stage relative to asymptomatic infectivity.	m_{Ss}	0.14	(14, 18–20)
F	Infectivity of hospitalized stage relative to asymptomatic infectivity.	m_H	0.07	(14, 18–20)
F	Infectivity of tested mild symptomatic stage relative to asymptomatic infectivity.	m_{YSm}	0.28	(14, 18–20)
F	Infectivity of tested severe symptomatic stage relative to asymptomatic infectivity.	m_{YSs}	0.14	(14, 18–20)
F	Infectivity of tested asymptomatic stage relative to asymptomatic infectivity.	m_{YA}	0.2	(14, 18–20)
F	Duration in days of incubation phase	$\frac{1}{\nu}+\frac{1}{\gamma_A+\gamma_S}$	5	(14, 21–23)

Continues on following page

Table S3, continued

Set	Parameter	Formula or Symbol	Value	Sources
F	Infectious duration in days of asymptomatic and mild disease	$\frac{1}{\xi_A}$	5	(14, 22–25)
F	Duration in day from first developing severe symptoms to being hospitalized. The development of critical symptoms (e.g., ARDS) is assumed to happen after one additional day.	$1/h$	0.1	(14, 26)
F	Expected days spent in hospital (including ICU) at hospitalization	$\frac{1}{\xi_H+\mu_H+A_{ICU}\chi}\left(1+\frac{\chi}{\xi_{ICU}+\mu_{ICU}}\right)$	8	(14, 26)
F	Proportion of incubation phase which is non-infectious	$\frac{\gamma_A+\gamma_S}{\gamma_A+\gamma_S+\nu}$	0.6	(22, 27)
F	Per person daily rate of seeking a test - assuming no capacity constraints	ζ	0.01	(2)
F	Per person daily detection rate for those who are symptomatic	ζ_S	0.1	(2)
F	Per person daily detection rate for those who are asymptomatic	ζ_A	0.01	(2)
F	Expected days spent in the ICU at ICU admission as a proportion of expected days spent in the hospital at hospitalization	$\frac{\xi_H+\mu_H+A_{ICU}\chi}{\xi_{ICU}+\mu_{ICU}+\chi}$	0.9	(14, 26)
F	Vaccine Overall Efficacy	e_v	0.95	(28, 29)
F	Average number of days to adjust NPI Levels.	l	14	(2)
F,D	Change in transmissibility from baseline value	$\Delta_\lambda(t)$	0	assumed
F,D	Increase in mixing due to vaccination	m_v	1	assumed
F,D	Maximum Vaccination Rate	$s_v^{\{\max\}}$	$3 * 10^{-3}$	assumed
F,D	Vaccine Transmission Efficacy	e_{tv}	1	assumed
F,D	Months before loss of natural immunity	ρ_N	20	(30)
F,D	Proportion of the Population willing to vaccinate.	W	0.9	(2)

Most of our parameters are defined based on clinical evidence. Because disease duration parameters are not informed by our calibration procedure (deaths time-series do not carry information about these rates) and to reduce the dimensionality of the calibration problem, we fix disease duration parameters that can be regarded as less uncertain at this stage in the pandemic. We also fix parameters that define the relative infectiousness of different disease states. Our prior work has established that these parameters have a small influence on the model outcomes

we use for calibration when one accounts for the ranges of the other, more uncertain and influential parameters (2).

Other parameters are more uncertain and benefit from calibration. For parameters unique to our model (i.e., the effectiveness of NPIs θ) or parameters poorly characterized from existing literature (i.e., magnitude of the seasonal effect on mixing in California during 2020), we use one of two approaches. The first approach is to provide prior ranges and let the calibration algorithm find combinations of parameters that jointly are consistent with the data. The NPI Effectiveness parameter θ and the behavioral adaptation factor b_h (to what extent people adapted after the initial lockdowns) are part of this set of parameters. Finally, there are parameters that are regarded as deep uncertainties. Further discussion about how these parameters enter our model is available in our prior work (2). The next section describes how the full experimental design is created using these parameters.

Experimental Design

Our full experimental design table is composed by the combination of the set of 78 strategies described in the Methods section and listed in Appendix I, the 100 calibration parameters vectors obtained using the calibration approach described earlier and a set of 200 draws from a Latin Hypercube sample of the six deeply uncertain parameters described in table S4. Instead of adding these deep uncertainties to the calibration process, we use baseline values during calibration and explored the uncertainty in their values after calibration. Our approach resembles the scenarios used by the Scenarios Hub initiative (31). However, instead of defining four discrete scenarios combining uncertainties (e.g. increased transmissibility driven by new variants) and decisions (e.g. relaxation of NPIs), we explore a wider set of strategies under a continuum of plausible futures. Table S4 presents each uncertainty explored in this analysis, the baseline value used during model calibration, and the minimum and maximum values. The baseline value reflects the assumptions commonly made by modelers elsewhere (e.g., there is no endogenous change in transmissibility driven by higher vaccination coverage).

Table S4. Deep Uncertainties and their ranges.

Uncertainty	Symbol	Baseline	Min	Max
Change in transmissibility from baseline value	$\Delta_\lambda(t)$	0	-25%	50%
Increase in mixing due to vaccination	m_v	0	0	20%
Maximum Vaccination Rate	$s_v^{\{\max\}}$	0.0030	0.0023	0.0038
Vaccine transmissibility efficacy factor	e_{tv}	100%	10%	100%
Average duration of immunity (months)	ρ_N	20	10	40
Proportion of the Population willing to vaccinate.	W	90%	54%	90%

All these uncertainties are applied to the model as an exogenous shift in the level of the baseline uncertainty values after the calibration period. Other functional forms, (e.g., smooth transitions from the baseline value to the new value) could also be implemented to represent these uncertainties with more realism. For example, modeling the impact of variant strains on transmissibility could be done with a logistic growth curve. However, considering these uncertainties using smooth transition functions would require an even larger experimental design to accommodate the additional parameters that control the rate and timing of the changes in parameters, which could be uncertain themselves. To keep our experimental design computationally tractable, we explore uncertainties by changing their values immediately after the calibration phase.

These uncertainties were chosen to include factors often considered by other modelers. For example, at the time of this writing, the MIDAS Network Scenario Hub uses changes in transmissibility driven by variants and vaccination uptake and efficacy (31). Our uncertainties were chosen to encompass these scenarios and to further explore the impacts of other concerns not commonly addressed in the existing literature. For example, uncertainties in behaviors related to vaccination include the actual maximum vaccination rate that society will achieve, the fraction of the population willing to vaccinate (applied to each population strata), and a potential increase in mixing after vaccination which is often ignored.

When multiple uncertainties are mechanistically entangled in our model (e.g., increases in transmissibility driven by new variant strains can be potentially offset by higher levels of mask-wearing), we use a single parameter that

represents the overall change in that parameter from baseline. For example, the change in transmissibility from a baseline value uncertainty $\Delta_\lambda(t)$ is intended to represent the combined effect of more transmissible variant strains (32), increased use of adaptation measures (such as reopening schools with enhanced mitigation protocols), as well as potential changes in the overall mixing. Because all these factors would affect the same transmission equation in our model, sampling multiple uncertainties in a Latin Hypercube to represent them separately would prove inefficient and unnecessary to our purposes. Therefore, we use a single uncertainty parameter to represent the combined effect of these factors.

After calibration, we construct our final experimental design as follows. As described in the calibration section, we obtain a weighted sample from the posterior containing 100 calibration parameter vectors. This sample is obtained with substitution, and resulted in 75 unique parameter sets. We do not need to spend computation time on duplicated parameter vectors. Therefore, we obtain the experimental design by combining the 200 parameter vectors obtained from the uncertainties, with the 75 unique parameter vectors obtained from the calibration procedure. This process results in 15,000 futures under which we test each reopening strategy. We obtain our full experimental design by randomizing the order of the 15,000 futures[†] and combining this resulting dataset with the set of 78 strategies, which resulted in 1.17 million unique cases to be run. After this process, we re-create the full experimental design by repeating the 25 non-unique calibration parameter vectors according to the number of times they were sampled from the posterior distribution.

The set of decisions we made with respect to our parameters in this particular analysis should not be interpreted as a set-in-stone representation of the pandemic. The set of uncertainties we chose, their bounds and how they were modeled represented our knowledge and concerns with respect to the pandemic in the US as of February 2021. In a regular Robust Decision Making engagement, this set of uncertainties would evolve as more information become available. Parameters that were once deep uncertainties would become regular calibration parameters. For example, if it becomes clear that vaccine prevents transmission, that parameter could be either set during the calibration phase or could be calibrated to data. Similarly, if highly transmissible variant strains become dominant, that change could be reflected in our model with a smooth function. When it becomes clear what will be the final vaccination rate, this parameter could be set. At that point, other uncertainties or policy options might emerge as important, and another analytical cycle could be undertaken to provide further results. The RDM iterative approach would accommodate these new developments, and decision-makers could regularly re-evaluate the robustness of their decisions to the remaining uncertainties of concern.

This section described the additions made to our original model, documented the parameters used in this analysis, and provided details on how the calibration parameters are used with the deeply uncertain parameters and strategies to create our large experimental design that is the basis of our conclusions. Further details about our model can be obtained from our prior work (1, 2).

Computing Environment

The calibration process and the strategy stress-testing runs were performed on Bebop, a High-Performance Computing cluster managed by the Laboratory Computing Resource Center at Argonne National Laboratory. Bebop has 1024 nodes comprised of 672 Intel Broadwell processors with 36 cores per node and 128 GB of RAM and 372 Intel Knights Landing processors with 64 cores per node and 96 GB of RAM. This analysis used slurm's array jobs to execute the runs in parallel across Broadwell nodes, using 35 cores per node and up to three jobs of 12 nodes at a time. The 1.17 million unique model runs used approximately 50,000 hours of CPU time to complete.

Code

The model and the functions used to perform this analysis were implemented in R. We developed the c19randepimod R package specifically to inform policies during the COVID-19 pandemic. The package includes a series of functions to gather data, define a c19model model class, calibrate the model, define experimental designs, run experiments and generate results. By defining our model as a class, we allowed ourselves and future users to extend our original c19model. Currently, models based on the c19model class necessarily need to be compatible with the deSolve package, but future versions can relax this requirement and allow stochastic models or ABMs. This allows us to create new model classes that inherit the functions implemented for the c19model class regardless of the model structure. For example, our original state policy tool published in May 2020 did not include vaccination, behavioral responses to vaccination and hesitancy, seasonality, and increases in transmissibility from variants, but the model used in this paper does. This flexibility and model design choices proved to be helpful and instrumental for our work. Readers can find our code and instructions to reproduce our work at https://github.com/RANDCorporation/covid-19-reopening-california.

[†] This is useful because it allows us to run a fraction of our experiments and evaluate results in the interim, before spending the 50,000 hours of computing time required to run the full experimental design.

References

1. R Vardavas, et al., The Health and Economic Impacts of Nonpharmaceutical Interventions to Address COVID-19: A Decision Support Tool for State and Local Policymakers. (2020).
2. R Vardavas, P Nascimento de Lima, L Baker, Modeling COVID-19 Nonpharmaceutical Interventions: Exploring periodic NPI strategies. *RAND Work. Pap.* (2021).
3. J Mossong, et al., Social Contacts and Mixing Patterns Relevant to the Spread of Infectious Diseases. *PLOS Medicine* **5**, e74 (2008).
4. K Prem, AR Cook, M Jit, Projecting social contact matrices in 152 countries using contact surveys and demographic data. *PLOS Comput. Biol.* **13**, e1005697 (2017).
5. P van den Driessche, J Watmough, Reproduction numbers and sub-threshold endemic equilibria for compartmental models of disease transmission. *Math. Biosci.* **180**, 29–48 (2002).
6. SY Lee, M Park, Y Shin, Hit Harder, Recover Slower? Unequal Employment Effects of the COVID-19 Shock. *NBER Work. Pap. Ser.* **53**, 1689–1699 (2021).
7. A Strong, J Welburn, An Estimation of the Economic Costs of Social-Distancing Policies. *An Estim. Econ. Costs Social-Distancing Policies* (2020).
8. K Shea, et al., COVID-19 reopening strategies at the county level in the face of uncertainty: Multiple Models for Outbreak Decision Support (2020).
9. CH Hansen, D Michlmayr, SM Gubbels, K Mølbak, S Ethelberg, Assessment of protection against reinfection with SARS-CoV-2 among 4 million PCR-tested individuals in Denmark in 2020: a population-level observational study. *The Lancet* **397** (2021).
10. CM Rutter, J Ozik, M Deyoreo, N Collier, Microsimulation model calibration using incremental mixture approximate bayesian computation. *Annals Appl. Stat.* **13**, 2189–2212 (2019).
11. EA Meyerowitz, A Richterman, II Bogoch, N Low, M Cevik, Towards an accurate and systematic characterisation of persistently asymptomatic infection with SARS-CoV-2. *The Lancet Infect. Dis.* **0** (2020).
12. O Byambasuren, et al., Estimating the extent of asymptomatic COVID-19 and its potential for community transmission: Systematic review and meta-analysis. *Off. J. Assoc. Med. Microbiol. Infect. Dis. Can.* **5**, 223–234 (2020).
13. D Buitrago-Garcia, et al., Occurrence and transmission potential of asymptomatic and presymptomatic SARS-CoV-2 infections: A living systematic review and meta-analysis. *PLoS medicine* **17**, e1003346 (2020).
14. CDC, COVID-19 Pandemic Planning Scenarios (https://www.cdc.gov/coronavirus/2019-ncov/hcp/duration-isolation.html) (2020).
15. SM Abate, SA Ali, B Mantfardo, B Basu, Rate of Intensive Care Unit admission and outcomes among patients with coronavirus: A systematic review and Meta-analysis. *PLOS ONE* **15**, e0235653 (2020).
16. LI Horwitz, et al., Trends in COVID-19 Risk-Adjusted Mortality Rates. *J. Hosp. Medicine* **16**, 90–92 (2021).
17. RA Armstrong, AD Kane, TM Cook, Outcomes from intensive care in patients with COVID-19: A systematic review and meta-analysis of observational studies. *Anaesthesia* **75**, 1340–1349 (2020).
18. A Goyal, EF Cardozo-Ojeda, JT Schiffer, Potency and timing of antiviral therapy as determinants of duration of SARS CoV-2 shedding and intensity of inflammatory response. *medRxiv*, 2020.04.10.20061325 (2020).
19. TC Quinn, et al., Viral load and heterosexual transmission of human immunodeficiency virus type 1. Rakai Project Study Group. *The New Engl. J. Medicine* **342**, 921–929 (2000).
20. X He, et al., Temporal dynamics in viral shedding and transmissibility of COVID-19. *Nat. Medicine* **26**, 672–675 (2020).
21. Wj Guan, et al., Clinical Characteristics of Coronavirus Disease 2019 in China. *New Engl. J. Medicine* **382**, 1708–1720 (2020).
22. C Rhee, S Kanjilal, M Baker, M Klompas, Duration of SARS-CoV-2 Infectivity: When is it Safe to Discontinue Isolation? *Clin. Infect. Dis. An Off. Publ. Infect. Dis. Soc. Am.* (2020).
23. AW Byrne, et al., Inferred duration of infectious period of SARS-CoV-2: Rapid scoping review and analysis of available evidence for asymptomatic and symptomatic COVID-19 cases. *BMJ Open* **10** (2020).
24. MC Kim, et al., Duration of Culturable SARS-CoV-2 in Hospitalized Patients with Covid-19. *New Engl. J. Medicine* **0**, null (2021).
25. CDC, Coronavirus Disease 2019 (COVID-19) (https://www.cdc.gov/coronavirus/2019-ncov/hcp/planning-scenarios.html) (2020).
26. C Faes, et al., Time between Symptom Onset, Hospitalisation and Recovery or Death: Statistical Analysis of Belgian COVID-19 Patients. *Int. J. Environ. Res. Public Heal.* **17** (2020).
27. L Zou, et al., SARS-CoV-2 Viral Load in Upper Respiratory Specimens of Infected Patients. *New Engl. J. Medicine* **382**, 1177–1179 (2020).

28. I ModernaTX, International Council for Harmonisation (ICH) Technical Requirements for Registration of Pharmaceuticals for Human Use, E6(R2) Good Clinical Practice (GCP) Guidance. *Mod. Inc* (2020).
29. FP Polack, et al., Safety and Efficacy of the BNT162b2 mRNA Covid-19 Vaccine. *The New Engl. journal medicine* (2020).
30. JS Lavine, ON Bjornstad, R Antia, Immunological characteristics govern the transition of COVID-19 to endemicity. *Sci. (New York, N.Y.)* (2021).
31. MIDAS Network, Announcing the new Scenario Modeling Hub (2020).
32. N Castonguay, W Zhang, MA Langlois, Meta-Analysis of the Dynamics of the Emergence of Mutations and Variants of SARS-CoV-2. *medRxiv* (2021).
